# Hyaluronidase-2 Regulates RhoA Signaling, Myofibroblast Contractility, and Other Key Profibrotic Myofibroblast Functions

**DOI:** 10.1016/j.ajpath.2020.02.012

**Published:** 2020-06

**Authors:** Adam C. Midgley, Emma L. Woods, Robert H. Jenkins, Charlotte Brown, Usman Khalid, Rafael Chavez, Vincent Hascall, Robert Steadman, Aled O. Phillips, Soma Meran

**Affiliations:** ∗ Wales Kidney Research Unit, Systems Immunity URI, Division of Infection and Immunity, College of Biomedical and Life Sciences, Cardiff University, Cardiff, United Kingdom; † Department of Biomedical Engineering, Lerner Research Institute, Cleveland Clinic, Cleveland, Ohio

## Abstract

Hyaluronidase (HYAL)-2 is a weak, acid-active, hyaluronan-degrading enzyme broadly expressed in somatic tissues. Aberrant HYAL2 expression is implicated in diverse pathology. However, a significant proportion of HYAL2 is enzymatically inactive; thus the mechanisms through which HYAL2 dysregulation influences pathobiology are unclear. Recently, nonenzymatic HYAL2 functions have been described, and nuclear HYAL2 has been shown to influence mRNA splicing to prevent myofibroblast differentiation. Myofibroblasts drive fibrosis, thereby promoting progressive tissue damage and leading to multimorbidity. This study identifies a novel HYAL2 cytoplasmic function in myofibroblasts that is unrelated to its enzymatic activity. In fibroblasts and myofibroblasts, HYAL2 interacts with the GTPase-signaling small molecule ras homolog family member A (RhoA). Transforming growth factor beta 1–driven fibroblast-to-myofibroblast differentiation promotes HYAL2 cytoplasmic relocalization to bind to the actin cytoskeleton. Cytoskeletal-bound HYAL2 functions as a key regulator of downstream RhoA signaling and influences profibrotic myofibroblast functions, including myosin light-chain kinase–mediated myofibroblast contractility, myofibroblast migration, myofibroblast collagen/fibronectin deposition, as well as connective tissue growth factor and matrix metalloproteinase-2 expression. These data demonstrate that, in certain biological contexts, the nonenzymatic effects of HYAL2 are crucial in orchestrating RhoA signaling and downstream pathways that are important for full profibrotic myofibroblast functionality. In conjunction with previous data demonstrating the influence of HYAL2 on RNA splicing, these findings begin to explain the broad biological effects of HYAL2.

Hyaluronan (HA) is a linear glycosaminoglycan, which is a ubiquitous component of extracellular matrix and has a major role in regulating cellular processes, such as cell–cell adhesion,^1^ migration,^2^, ^3^, ^4^ differentiation,^5^^,^^6^ and proliferation.^7^, ^8^, ^9^ HA is therefore implicated in influencing numerous biological processes, and dysregulation of HA synthesis, turnover, and binding interactions contributes to a multitude of disease states, such as atherosclerosis, chronic inflammation, cancer progression, and fibrosis.^10^, ^11^, ^12^, ^13^

Hyaluronidase (HYAL)-2 has been identified as one of the principal enzymes involved in HA catabolism in vertebrates. HYAL2 is broadly expressed in tissues but has catabolic function within only a narrow acidic pH range (optimal pH, 4), and compared to other HYALs, has only weak intrinsic HA-degrading activity.^14^ HYAL2 was originally identified as a lysosomal enzyme but was also subsequently identified as anchored to the cell membrane via a glycosylphosphatidylinositol link.^15^ Aberrant expression of HYAL2 is implicated in diverse pathology, including cardiac and skeletal abnormalities, hematopoietic and platelet dysfunction, cancer, and fibrosis.^16^, ^17^, ^18^, ^19^, ^20^, ^21^ However, many reports indicate that a significant proportion of expressed HYAL2 may be enzymatically inactive; thus the cellular function of HYAL2 and the mechanisms through which HYAL2 dysregulation influences pathology have been previously unclear.^14^^,^^15^^,^^22^ A number of studies have identified that HYAL2 can also have important nonenzymatic functions: Glycosylphosphatidylinositol-anchored HYAL2 has been identified as acting as a co-receptor for the transmembrane glycoprotein CD44, as a regulator of transforming growth factor (TGF)-β1–mediated intracellular WW domain–containing oxidoreductase 1 signaling, and as a viral entry receptor.^23^, ^24^, ^25^, ^26^ More recently, it was determined that glycosylphosphatidylinositol-anchored HYAL2 can translocate to the nucleus and regulate alternative splicing events that influence differentiation to profibrotic cell phenotypes.^27^

Myofibroblasts are the principal effector cells that drive progressive fibrosis, a process that underlies many organ-specific diseases and contributes to the burden of multimorbid conditions, including chronic kidney disease, lung fibrosis, liver cirrhosis, and degenerative joint disease.^28^, ^29^, ^30^, ^31^, ^32^, ^33^ Therefore, the study of factors that can either promote or prevent cell differentiation to a myofibroblast phenotype is important in identifying new therapeutic approaches to the treatment of chronic disease. Myofibroblasts are derived from differentiation of resident fibroblasts, pericytes, or epithelial cells under the influence of circulating profibrotic cytokines, such as TGF-β1.^13^^,^^34^, ^35^, ^36^ HA and the most widely expressed isoform of the HA receptor, CD44, are key mediators of myofibroblast differentiation.^37^, ^38^, ^39^, ^40^, ^41^, ^42^ Specifically, the presence of pericellular HA matrices tethered to cell-surface CD44 are essential for TGF-β1–driven myofibroblast differentiation. In contrast, cell-surface expression of an alternatively spliced variant isoform of CD44 (denoted CD44v7/8) promotes prevention and/or reversal of TGF-β1–driven myofibroblast differentiation by causing internalization of pericellular HA matrices.^27^^,^^43^ Nuclear HYAL2 was identified as a key modulator of *CD44* mRNA alternative splicing leading to attenuated standard *CD44* expression, while augmenting CD44v7/8 splice variant expression.

The purpose of this study was to determine the function of HYAL2 in myofibroblasts relevant to its cell localization. We report that cytoplasmic HYAL2 has distinct functions compared to nuclear HYAL2. In contrast to our previous studies demonstrating the antifibrotic actions of nuclear HYAL2,^27^ we show that cytoplasmic HYAL2 in myofibroblasts can bind to the actin cytoskeleton and function as a master regulator of TGF-β1–driven RhoA signaling. Through this, HYAL2 promotes key profibrotic myofibroblast functions, including myofibroblast contractility, collagen deposition, and *CTGF* (*CCN2*) and *MMP2* mRNA expression. These emerging studies from our group and others begin to explain the broad biological effects of HYAL2 and identify it as a key molecule for future study in chronic disease.

## Materials and Methods

### Materials

All reagents were purchased from Sigma-Aldrich (Poole, UK) or Thermo Fisher Scientific (Paisley, UK) unless otherwise stated. Reverse-transcription reagents, siRNA transfection reagents, and real-time quantitative PCR (qPCR) primers and reagents were purchased from Thermo Fisher Scientific. Other reagents used were recombinant human TGF-β1 (R&D Systems, Abingdon, UK) and the RhoA inhibitor Rhosin (G04; Merck Millipore, Watford, UK).

### Animal Experiments Using Ischemia Reperfusion Injury–Induced Renal Fibrosis

Ten adult (8-week–old to 12-week–old) male Lewis rats weighing 180 to 220 g were used (Harlan Laboratories, Ltd., Derby, UK). The rats acclimated to their surroundings for 7 days, with housing, handling, and experimental procedures in accordance with the local institutional policies and procedures licensed by the UK Home Office under the Animals (Scientific Procedures) Act (1986). Rats (*n* = 5 in each treatment group) were provided with analgesics (200 μg of buprenorphine dissolved in 500 mL of drinking water) from 24 hours before surgery until kidney retrieval. Animals were anesthetized with isoflurane, a midline laparotomy incision made, and the renal pedicles were identified and clamped for 45 minutes using a vascular clip (ischemia reperfusion injury group). The kidney was visually assessed for both ischemia upon clamping and reperfusion upon release of the clamp. Rats in the sham group underwent the same operation without renal pedicle clamping (*n* = 5). The rats were maintained for 28 days in accordance with local institutional policies and procedures. At 28 days, kidney tissue was retrieved with the animals under terminal anesthesia and stored in formalin. Kidneys were later embedded in paraffin and sections of 4 μm in thickness were cut. Sections were deparaffinized and rehydrated using xylene and reducing concentrations of ethanol. Antigen retrieval was performed in an autoclave using sodium citrate buffer with Tween. After blocking of nonspecific sites, sections were incubated with 6 μg/mL anti-HYAL2 (goat anti-human polyclonal species reactivity includes rat; Abcam, Cambridge, UK) and anti–α-smooth muscle actin (SMA) monoclonal antibody 1A4 (mouse anti-human species reactivity includes rat; Thermo Fisher Scientific). Fluorescence-labeled secondary antibodies used were donkey anti-goat (H + L) Alexa Fluor 555 (Thermo Fisher Scientific) for HYAL2 and goat anti-mouse IgG (H + L) Alexa Fluor 488 (Thermo Fisher Scientific) for α-SMA. DAPI was used for nuclear staining and sections were analyzed using laser scanning confocal microscopy.

### Cell Culture

Human lung fibroblasts (AG02262) were purchased from Coriell Cell Repositories (Coriell Institute for Medical Research, Camden, NJ). The cells were cultured in Dulbecco's modified Eagle's low-glucose medium and Ham's F-12 containing 5 mmol/L glucose, 2 mmol/L l-glutamine, 100 U/mL penicillin, and 100 μg/mL streptomycin, and supplemented with 10% fetal bovine serum (Biological Industries Ltd., Cumbernauld, UK). The cells were maintained at 37°C in a humidified incubator in an atmosphere of 5% CO_2_, and fresh growth medium was added to the cells every 3 days until the cells were ready for experimentation. The cells were incubated in serum-free medium for 48 hours before use in all experiments (growth arrest), and all experiments were performed under serum-free conditions unless otherwise stated. All experiments were undertaken using cells at passages 6 to 10.

### RT-PCR and qPCR

qPCR was used to assess mRNA expression levels of α-SMA (*ACTA2*), hyaluronidase 2 (HYAL2), extra domain-A–fibronectin (*EDA-FN*), transforming growth factor beta 1 (*TGFB1*), [cellular communication network factor 2 (*CCN2*), also known as connective tissue growth factor (CTGF)], fibronectin (*FN1*), collagen I [collagen type I alpha 1 chain (*COL1A1*)] and collagen type I alpha 2 chain (*COL1A2*), and matrix metallopeptidase 2 (*MMP2*). Primers were commercially designed and purchased from Thermo Fisher Scientific (Table 1). The cells were grown in 35-mm dishes and washed with phosphate-buffered saline (PBS) before lysis with TRI Reagent and RNA purification according to the manufacturer's protocol. Reverse-transcription used high-capacity cDNA reverse-transcription kits, according to the manufacturer's protocols (Thermo Fisher Scientific). The kits use the random primer method for initiating cDNA synthesis. As a negative control, reverse-transcription was done with RNase-free, sterile H_2_O replacing the RNA sample. qPCR was done using the ViiA 7 Real-Time qPCR System (Thermo Fisher Scientific) in a final volume of 20 μL per sample, as follows: 1 μL of reverse-transcription product, 0.6 μL of target gene forward primer, 0.6 μL of target gene reverse primer, 10 μL of Power SYBR Green PCR Master Mix, and 7.8 μL of sterile RNase-free water. Amplification was done using a cycle of 95°C for 15 seconds and 60°C for 1 minute for 40 cycles, followed by a melt-curve stage at 95°C for 15 seconds, 60°C for 1 minute, and a final step of 95°C for 15 seconds. qPCR was simultaneously performed for *GAPDH* (primers and probe commercially designed and purchased from Thermo Fisher Scientific) as a standard reference gene. As a negative control, qPCR was performed with nuclease-free, sterile H_2_O replacing the cDNA sample. The comparative *C*_τ_ method was used for relative quantification of gene expression. The *C*_τ_ (threshold cycle where amplification is in the linear range of the amplification curve) for the standard reference gene (*GAPDH*) was subtracted from the target gene *C*_τ_ to obtain the Δ*C*_τ_. The mean Δ*C*_τ_ values for replicate samples were then calculated. The expression of the target gene in experimental samples relative to expression in control samples was then calculated using the following equation: 2^−[Δ*C*τ(1)−Δ*C*τ(2)]^, where Δ*C*_τ_(1) is the mean Δ*C*_τ_ calculated for the experimental samples, and Δ*C*_τ_(2) is the mean Δ*C*_τ_ calculated for the control samples.

**Table 1:** Primer Sets Used in Quantitative RT-PCR Experiments

Target	Sequences
*HYAL2*	F: 5′-CGGACTCCCACACAGTTCCT-3′ R: 5′-CCAGGGCCAATGTAACGGT-3′
*EDA-FN*	F: 5′-GCTCAGAATCCAAGCGGAGA-3′ R: 5′-CCAGTCCTTTAGGGCGATCA-3′
*TGFB1*	F: 5′-CCTTTCCTGCTTCTCATGGC-3′ R: 5′-ACTTCCAGCCGAGGTCCTTG-3′
*CCN2*	F: 5′-GGCCCAGACCCAACTATGAT-3′ R: 5′-AGGCGGCTCTGCTTCTCTA-3′
*FN1*	F: 5′-CCGAGGTTTTAACTGCGAGA-3′ R: 5′-TCACCCACTCGGTAAGTGTTC-3′
*MMP2*	F: 5′-CGTCGCCCATCATCAAGTTC-3′ R: 5′-CAGGTATTGCACTGCCAACTC-3′
*COL1A1*	F: 5′-TGTTCAGCTTTGTGGACCTCCG-3′ R: 5′-CGCAGGTGATTGGTGGGATGTCT-3′
*COL1A2*	F: 5′-GGCTCTGCGACACAAGGAGT-3′ R: 5′-TGTAAAGATTGGCATGTTGCTAGGC-3′
*GAPDH*	F: 5′-CCTCTGACTTCAACAGCGACAC-3′ R: 5′-TGTCATACCAGGAAATGAGCTTGA-3′
*ACTA2*	TaqMan assay gene ID Hs00426835_g1 (Thermo Fisher Scientific, Paisley, UK)
18S rRNA	Product code 4310893E (Thermo Fisher Scientific)

F, forward; R, reverse.

### Immunocytochemistry

Cells were grown to 70% confluence in eight-well Permanox chamber slides. The culture medium was removed, and the cells washed with sterile PBS before fixation in 4% paraformaldehyde for 10 minutes at room temperature. After fixation, cells were permeabilized with 0.1% (v/v) Triton X-100 in PBS for 10 minutes at room temperature. Slides were blocked with 1% bovine serum albumin (BSA) for 1 hour before a further washing step with 0.1% (wt/v) BSA in PBS. Subsequently, the slides were incubated with the primary antibody diluted in 0.1% BSA and PBS for 2 hours at room temperature. After a further washing step, slides were incubated with Alexa Fluor 488–conjugated and/or Alexa Fluor 594–conjugated secondary antibodies for 1 hour at room temperature. Cell nuclei were stained with Hoechst solution. Cells were then mounted and analyzed by confocal and fluorescent microscopy. The following primary antibodies were used: mouse anti-human α-SMA (Sigma-Aldrich) and rabbit anti-human HYAL2 antibody (Atlas Antibodies, Sigma-Aldrich). The following secondary antibodies were used: goat anti-mouse Alexa Fluor 488, goat anti-rabbit Alexa Fluor 594 (InvitroGen/Thermo Fisher Scientific). For visualization of F-actin, fluorescein isothiocyanate–conjugated phalloidin toxin was used in place of primary antibodies (Sigma-Aldrich). The total HYAL2 intensity of expression was quantified, and localization was measured and quantified to regions of the cells using ImageJ software version 1.37c (NIH, Bethesda, MD; *http://imagej.nih.gov/ij*) and the Wright Cell Imaging Facility Intensity Correlation Analysis plug-in.

### siRNA Transfection

Fibroblasts were transiently transfected with specific siRNA nucleotides (Thermo Fisher Scientific) targeting HYAL2. Transfection was done using Lipofectamine 2000 transfection reagent (InvitroGen/Thermo Fisher Scientific) in accordance with the manufacturer's protocol. Briefly, cells were grown to 50% to 60% confluence in antibiotic-free medium in either 35-mm dishes or eight-well Permanox chamber slides. Transfection reagent (2% v/v) was diluted in Opti-MEM reduced growth medium (Gibco/Thermo Fisher Scientific) and left to incubate for 5 minutes at room temperature. *HYAL2* siRNA (si*HYAL2*) oligonucleotides were diluted in Opti-MEM reduced growth medium to achieve a final concentration of 30 nmol/L. The transfection reagent and siRNA mixtures were then combined and incubated at room temperature for an additional 20 minutes. The newly formed transfection complexes were subsequently added to the cells and incubated at 37°C with 5% CO_2_ for 24 hours, before replacement with fresh serum-free medium before experimentation. As a control, cells were transfected with negative-control siRNA (scramble; a sequence that bears no homology to the human genome) (Thermo Fisher Scientific).

### Overexpression Vector Generation and Transfection

The *HYAL1* or *HYAL2* open reading frame was inserted into the vector pCR3.1 using a standard ligation reaction with T4 DNA ligase (New England BioLabs, Hitchin, UK). Amplification of the cloned vector was performed via bacterial transformation into one-shot competent *Escherichia coli* (New England BioLabs) and grown overnight on ampicillin containing agar. Single colonies were extracted, cloned, and DNA-purified according to the Miniprep Kit protocol (Sigma-Aldrich). Cloned pCR3.1-*HYAL1* or pCR3.1-*HYAL2* vector uptake of the insert was confirmed with DNA sequencing. Transfection was done using Lipofectamine LTX transfection reagent (InvitroGen/Thermo Fisher Scientific) in accordance with the manufacturer's protocol. Briefly, cells were grown to 50% to 60% confluence in antibiotic-free medium in either 35-mm dishes or eight-well Permanox chamber slides. Transfection reagent (2% v/v) was diluted in Opti-MEM reduced growth medium (Gibco/Thermo Fisher Scientific). pCR3.1 overexpression vectors were diluted in Opti-MEM reduced growth medium containing PLUS Reagent (1% v/v), to achieve a final transfection concentration of 1.5 μg/mL. The transfection reagent and plasmid mixtures were then combined and incubated at room temperature for an additional 20 minutes. The newly formed transfection complexes were subsequently added to the cells and incubated at 37°C with 5% CO_2_ for 24 hours, before replacement with fresh serum-free medium before experimentation. As a control, cells were transfected with empty pCR3.1 plasmid (mock transfection plasmid containing no open reading frame cDNA). Negative RT experiments were performed alongside *HYAL1/HYAL2* mRNA PCR to ensure that the vectors were not conveying false-positive overexpression. Expression was confirmed by visualization on a 2% agarose gel.

### HA Substrate Gel Zymography

Confluent monolayers of fibroblasts were cultured in T25 culture flasks, growth-arrested, and incubated in serum-free medium containing 10 ng/mL TGF-β1 for up to 72 hours. The conditioned medium (CM) was removed, and the cells were extracted in lysis buffer. The CM and the cell lysate (CL) were passed over DEAE-Sephacel ion-exchange columns in 50 mmol/L Tris-HCl, pH 7.8. The flow-through containing the HYAL enzymes was collected, dialyzed against H_2_O, and lyophilized. The samples were reconstituted in 100 mL of H_2_O and mixed with an equal volume of zymography loading buffer. Samples were loaded onto SDS-free 7.5% polyacrylamide gels containing HA at a final concentration of 0.17 mg/mL. Positive controls consisted of 1 mL of human serum diluted in 10 mL of H_2_O, mixed with an equal volume of zymography loading buffer. Electrophoresis was performed in running buffer at 75 V for 3 hours on ice to prevent denaturation of the HYAL enzymes. The gel was incubated in 0.1 mol/L sodium formate buffer, pH 3.7 at 37°C for 72 hours. The gel was stained with 0.5% (wt/v) Alcian Blue and then with 0.1% (w/v) Coomassie Brilliant Blue in 50% (v/v) methanol and 20% (v/v) acetic acid. The gel was destained in 5% (v/v) methanol and 10% (v/v) acetic acid. The gel was visualized and imaged using a light-box.

### Purification of [^3^H]-HA

Hexokinase-2 cells were used to prepare large quantities of [^3^H]-HA for analysis of HYAL enzyme activity. Cells were incubated with D-[^3^H]-glucosamine for 72 hours in serum-free medium. The CM was removed and the [^3^H]-HA isolated and subjected to size-exclusion chromatography on a Sephacryl S-500 column equilibrated with 4 mol/L guanidine buffer. The fractions corresponding to [^3^H]-HA, with a size of >1000 kDa, were pooled. Detergent was removed from [^3^H]-HA by passing over DEAE-Sephacel ion-exchange columns and washing bound [^3^H]-HA extensively with water. The [^3^H]-HA was eluted with detergent-free 4 mol/L guanidine buffer. For HYAL activity assays (50 × 10^3^ dpm) [^3^H]-HA was dialyzed against H_2_O and lyophilized.

### Hyaluronidase Activity Assay

Confluent monolayers of fibroblasts were cultured in T25 culture flasks, growth-arrested and incubated in serum-free medium containing 10 ng/mL TGF-β1 for up to 72 hours. The CM was removed, and the cells were extracted in lysis buffer. The CM and the CL were passed over DEAE-Sephacel ion-exchange columns in 50 mmol/L Tris-HCl, pH 7.8. The flow-through containing the HYAL enzymes was collected, dialyzed against H_2_O, and lyophilized. The samples were reconstituted in 100 mL of H_2_O and incubated with 200 mL of 0.1 mol/L sodium formate, pH 3.7 containing [^3^H]-HA (50 × 10^3^ dpm) for 72 hours. Controls consisted of 100 mL of H_2_O, 200 mL of 0.1 mol/L sodium formate buffer, pH 3.7 containing [^3^H]-HA (50 × 10^3^ dpm), incubated for 72 hours in the absence of CM or CL. The samples were mixed with an equal volume of 4 mol/L guanidine buffer and analyzed by size-exclusion chromatography on a Sephacryl S-500 column equilibrated with 4 mol/L guanidine buffer or a Sepharose CL-4B column (Amersham Pharmacia Biotech, Buckinghamshire, UK) equilibrated with 4 mol/L guanidine buffer. HYAL activity was assessed by comparing the [^3^H]-HA elution profiles, incubated in the presence and absence of CM or CL. HYAL activity was not detectable at neutral pH (data not shown).

### Co-Immunoprecipitation

Cells were harvested into 1 mL of ice-cold PBS and pelleted at 1000 g for 5 minutes before resuspension in radioimmunoprecipitation assay lysis buffer. Supernatant was transferred to new Eppendorfs and a known volume of 25 μg of protein in radioimmunoprecipitation assay buffer was immunoprecipitated using anti-HYAL2 antibody-linked anti-rabbit Dynabeads (InvitroGen/Thermo Fisher Scientific). Beads were preincubated with 0/1% w/v BSA. Immunoprecipitation was completed with an overnight rotating incubation at 4°C. After three washes with Nonidet P-40 buffer, the beads were resuspended in PBS and transferred to clean microcentrifuge tubes. The bead-antibody-protein complex was boiled with reducing buffer for 5 minutes before the supernatant was transferred into gel lanes for SDS-PAGE. Alternatively, eluted protein was assessed by mass spectrometry (MS).

### Mass Spectrometry

Co-immunoprecipitation elutes were loading into and run on a 1.5-mm 7.5% polyacrylamide gel. Gel plugs were manually excised and peptides recovered after trypsin (6.25 ng/mL in 25 mmol/L NH_4_HCO_3_, 37°C, 3 hours; sequencing grade–modified trypsin from Promega UK Ltd., Southampton, UK) digestion using a modified version of the method of Shevchenko et al.^44^ The dried peptides were resuspended in 50% (v/v) acetonitrile in 0.1% (v/v) trifluoroacetic acid (5 μL) for MS analysis and a 10% aliquot was spotted onto a 384-well MS plate. The samples were allowed to dry and were then overlaid with α-cyano-4-hydroxycinnamic acid [Sigma-Aldrich; 0.5 μL of 5 mg/mL in 50% (v/v) acetonitrile and 0.1% (v/v) trifluoroacetic acid]. MS was performed using a 4800 MALDI TOF/TOF mass spectrometer (Applied Biosystems, Thermo Fisher Scientific, Warringon, UK) with a 200-Hz solid-state laser operating at 355 nm (*S3*, *S4*). MALDI mass spectra and subsequent MS/MS spectra of the eight most abundant MALDI peaks were obtained after routine calibration. Peaks were stringently selected and were analyzed with the strongest peak first. For positive-ion reflector mode spectra 800 laser shots were averaged (mass range, 700 to 4000 Da; focus mass, 2000). In MS/MS positive ion mode 4000 spectra were averaged with 1 kV collision energy (collision gas was air at a pressure of 1.6 × 10^−6^ Torr) and default calibration. Combined peptide mass fingerprinting and MS/MS queries were performed using the Mascot database search engine version 2.1 (Matrix Science Ltd., London, UK) embedded into Global Proteome Server Explorer software version 3.6 (Applied Biosystems, Thermo Fisher Scientific) on the Swiss-Prot database (*https://www.uniprot.org*, last accessed January 9, 2013) or the TrEMBL database (*www.bioinfo.pte.hu/more/trembl.htm*, download date June 28, 2011).^45^ Searches were restricted to human taxonomy with trypsin specificity (one missed cleavage allowed), the tolerances were set for peptide identification searches at 50 ppm for MS and 0.3 Da for MS/MS. Cysteine modification by iodoacetamide was employed as a fixed modification with methionine oxidation as a variable modification. Search results were evaluated by manual inspection, and conclusive identification confirmed whether there were high-quality MS/MS (good y ion) data for two or more peptides (E value *P* < 0.05 for each peptide; overall *P* < 0.0025) or one peptide (only if the E value was *P* < 0.0001).

### Western Blot Analysis

Total protein was extracted in radioimmunoprecipitation assay lysis buffer containing 1% protease inhibitor cocktail, 1% phenylmethylsulfonyl fluoride, and 1% sodium orthovanadate (Santa Cruz Biotechnology, Santa Cruz, CA). Protein was quantified before SDS-PAGE and transfer to nitrocellulose. Membranes were blocked with 5% BSA/0.5% Tween-20/PBS for 1 hour, room temperature, followed by incubation with primary antibodies diluted in 1% BSA/0.1% Tween-20/PBS, overnight at 4°C. After wash steps, membranes were incubated in secondary anti-rabbit/mouse IgG horseradish peroxidase conjugate (Cell Signaling Technology, Beverly, MA; 1:5000 dilution, 1% BSA/0.1% Tween-20/PBS). Detection was performed using ECL reagent (GE Healthcare, Buckinghamshire, UK) and image exposure on a C-DiGit Western Blot Scanner (LI-COR, Bad Homburg vor der Höhe, Germany). Primary antibodies used were rabbit polyclonal to γ-actin (Abcam), rabbit polyclonal to phospho–myosin light-chain kinase (MLCK) S1760 (Abcam), mouse monoclonal to β-actin (Cell Signaling Technologies), mouse monoclonal anti–α-SMA antibody 1A4 (Thermo Fisher Scientific), and rabbit polyclonal anti-HYAL2 antibody (Sigma-Aldrich). Secondary antibodies used were goat polyclonal antibody to mouse IgG horseradish peroxidase (Abcam) and goat polyclonal antibody to rabbit IgG horseradish peroxidase (Abcam).

### *In Silico* Analysis

MS data were input into STRING (STRING database version 10.5; String Consortium 2017, *http://string-db.org*, last accessed July 1, 2017) and Kyoto Encyclopedia of Genes and Genomes (KEGG) pathway (KEGG Pathway database, *http://www.genome.jp/kegg*, last accessed February 20, 2020; Kanehisa Laboratories, Kyoto, Japan) analysis tools. STRING protein–protein networks indicate known protein–protein associations and strength of association, cross-referencing GO, InterPro and KEGG molecular pathways indicate strength of specific pathway involvement.^46^ KEGG pathway analysis was used to determine the functional pathway in which the proteins identified by MS were likely to be involved.

### Collagen Gel Contraction Assays

Type I collagen was extracted from rat-tail tendon as previously described.^47^ Approximately 2.5 × 10^5^ fibroblasts/mL were mixed into collagen lattice–forming solutions (2.5 mL 20% v/v fetal calf serum–Dulbecco's modified Eagle's low-glucose medium, 500 μL of 0.1 mol/L NaOH, and 2 mg/mL type I collagen; total volume of 5 mL). Fibroblast-populated collagen lattices (FPCLs) were maintained at 37°C, in a 5% CO_2_ atmosphere for 1 hour, for collagen polymerization to occur. FPCLs were gently detached from the plate edges and resuspended in serum-free medium containing appropriate treatments. FPCLs were measured at 0, 3, 6, 12, and 24 hours after initial lattice fabrication. The mean FPCL contraction values were obtained from analysis by ImageJ software version 1.37c (and are expressed as the percent reduction in gel diameter compared to the gel diameter at 0 days).

### Scratch-Wound Migration Assays

Scratching quiescent fibroblasts cell monolayers with sterile 200-μL pipette tips generated linear denuded areas. The cells were gently washed with PBS to remove detached cells and then replenished with fresh serum-free medium containing appropriate treatments. The wound size was photographed at 0, 3, 6, 12, and 24 hours or until closure, using an Axiovert 100 mol/L inverted microscope (Carl Zeiss, Oberkochen, Germany) fitted with a digital camera (Orca-1394; Hamamatsu Photonics K.K., Hamamatsu, Japan). Measurements were obtained using ImageJ software version 1.37c. Data are expressed as the percent reduction in wound area, compared to wound area at 0 hours.

### Statistical Analysis

The two-tailed, unpaired *t*-test was used to assess statistical differences between the two experimental groups. For experiments with multiple experimental groups, one-way analysis of variance was used to identify statistical differences across groups, followed by post-test Bartlett and multiple comparisons. For experiments with multiple experimental conditions, two-way analysis of variance was used, followed by post-test Tukey multiple comparisons. Graphical data are expressed as means ± SEM. All data were analyzed using GraphPad Prism software version 6 (GraphPad Software, San Diego, CA). *P* < 0.05 was considered statistically significant.

## Results

### Increased HYAL2 Expression and Colocalization with α-SMA–Positive Myofibroblasts in the Renal Interstitium after Experimental Renal Fibrosis *in Vivo*

**Figure 1:**
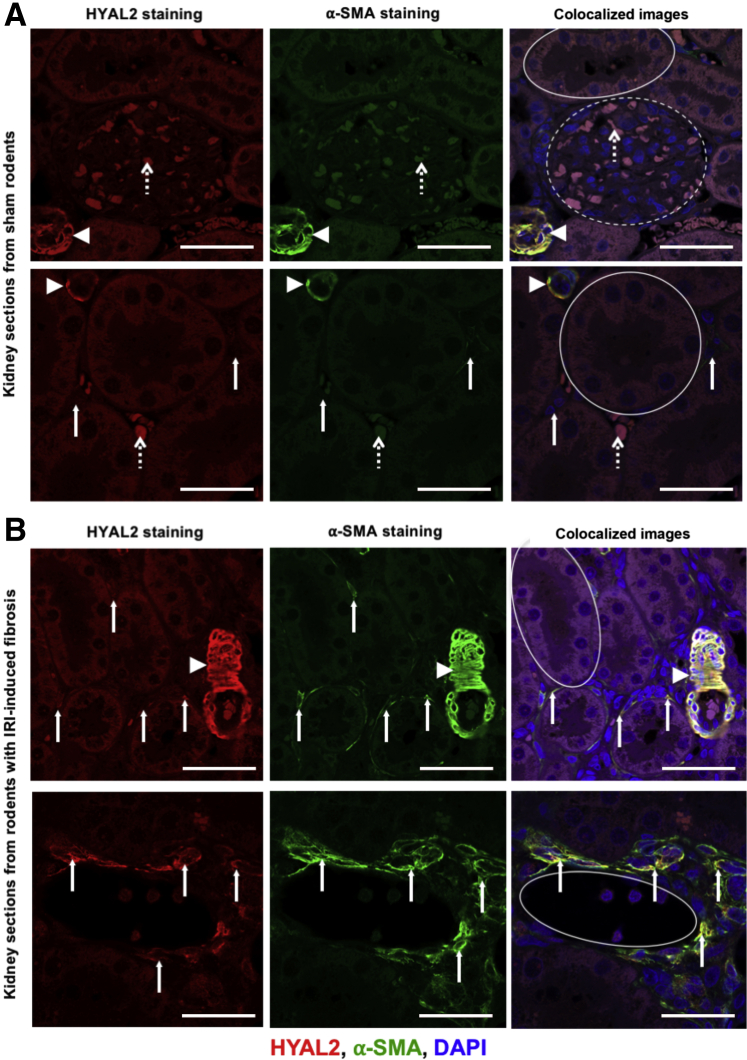
Hyaluronidase (HYAL)-2 demonstrates increased interstitial expression in renal fibrosis in areas of α-smooth muscle actin (SMA)–positive myofibroblasts. Adult male Lewis rats underwent a midline laparotomy and were divided into two groups: sham operation (**A**) and bilateral ischemia reperfusion injury (IRI) (cross-clamping of both renal pedicles for 45 minutes) (**B**). At 28 days postoperatively, kidney tissue was retrieved with the animals under terminal anesthesia and stored in formalin. Kidneys were later embedded in paraffin and sections of 4 μm in thickness were cut. Sections were deparaffinized and rehydrated using xylene and reducing concentrations of ethanol. Antigen retrieval was performed in an autoclave using sodium citrate buffer with Tween. After blocking of nonspecific sites, sections were incubated with anti-HYAL2 and α-SMA monoclonal antibodies with appropriate fluorescence-labeled secondary antibodies [HYAL2 Alexa Fluor 555 (red) and α-SMA Alexa Fluor 488 (green)] and DAPI nuclear staining (blue). Sections were analyzed using confocal microscopy. Areas depicted as yellow demonstrate areas of Hyal2 and α-Sma colocalization. **Dashed circle** indicates a glomerulus; **solid circles**, tubules; **arrowheads**, blood vessels; **dashed arrows**, areas of red blood cell autofluorescence to be disregarded; **solid arrows**, the renal interstitium between the tubules where fibroblasts and myofibroblasts reside. *n* = 5 per group. Scale bars = 50 μm. Original magnification, ×400 (**A** and **B**, **top rows**); ×630 (**A** and **B**, **bottom rows**).

To investigate the *in vivo* relevance of HYAL2 in progressive fibrosis, we characterized HYAL2 expression, localization, and preponderance in relation to the expression of the myofibroblast marker α-Sma in an experimental model of renal fibrosis (Figure 1). Our established model of bilateral renal ischemia was used to promote renal fibrosis in rats as described in the methods, and comparisons were made to sham controls. The results demonstrated that in normal/sham kidneys (Figure 1A), HYAL2 staining was present only in arterial blood vessels, where it colocalized with α-Sma staining from vascular smooth muscle cells. There was little/no HYAL2 staining in the glomeruli or in the tubules. There was some nonspecific HYAL2 staining of red blood corpuscles in the glomeruli and interstitium consistent red cell autofluorescence, which is a common phenomenon in the kidney. The renal interstitium is the space between the renal tubules and glomeruli where the fibroblasts/stromal cells reside. Resident fibroblasts were seen in the renal interstitium; however, these demonstrated no α-Sma expression, indicating that these cells had not differentiated to a profibrotic myofibroblast phenotype. The resident fibroblasts demonstrated little/no HYAL2 expression.

Kidneys harvested from rats that had undergone ischemia reperfusion injury–induced renal fibrosis (Figure 1B) also demonstrated positive HYAL2 staining in arterial blood vessels, and this similarly colocalized with α-Sma expression from vascular smooth muscle cells. However, in these kidney sections there was also clear evidence of increased expression of α-Sma–positive myofibroblasts in the renal interstitium. The areas of interstitial α-Sma–positive staining also demonstrated increased HYAL2 expression, which colocalized with α-Sma–positive areas. The top panel of images in Figure 1B (from rodents that underwent ischemia reperfusion injury) depicts an area of fibrosis with relatively preserved renal architecture with undamaged renal tubules. In these images there is α-Sma–positive myofibroblast staining with associated HYAL2 staining and colocalization of these two proteins in the renal interstitium. The lower panel of images in Figure 1B depicts an area of gross fibrosis with disordered renal tubular architecture and increased α-Sma–positive myofibroblast staining in the renal interstitium. These areas of enhanced fibrosis and damage also demonstrated increased levels of HYAL2 staining and colocalization with α-Sma–positive myofibroblasts. These data indicate that during progressive fibrosis, there is increased differentiation to and expansion of the α-SMA–positive myofibroblast cell population in the renal stroma, and that HYAL2 expression is increased in this cell population and colocalizes with α-SMA–positive cytoskeleton.

### Increased HYAL2 Expression in Myofibroblasts Associated with the Cell Cytoskeleton

**Figure 2:**
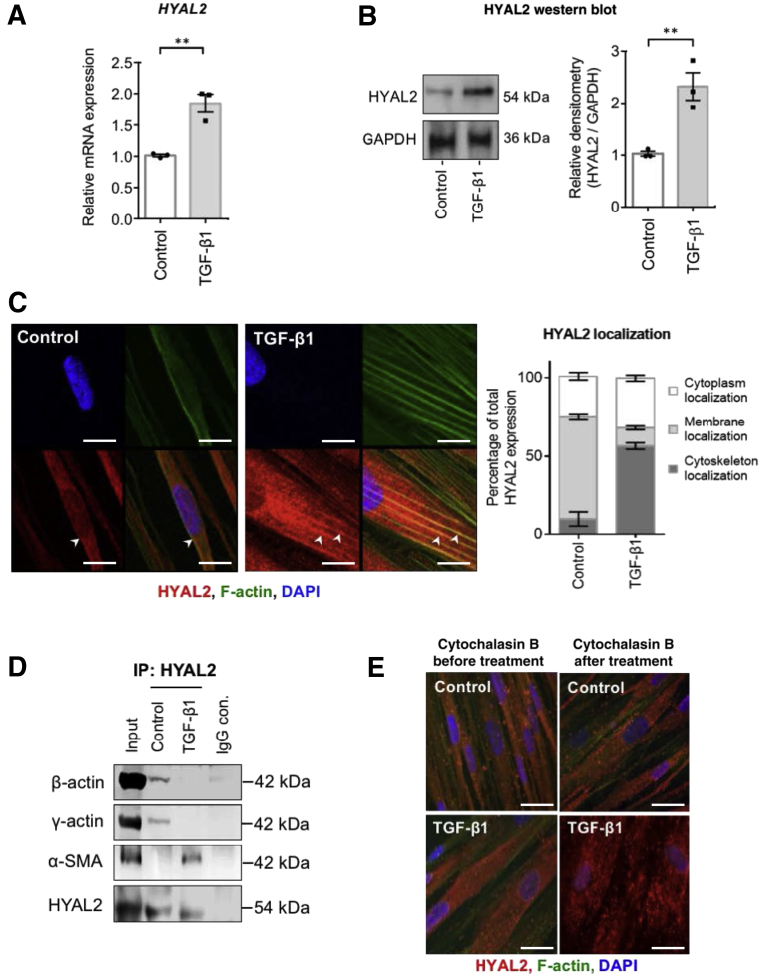
Hyaluronidase (HYAL)-2 is upregulated and relocated from the cell membrane to the cell cytoskeleton by transforming growth factor beta 1 (TGFB1) stimulation. Fibroblasts were seeded into 35-mm cell culture plates and grown to confluent monolayers. After 48 hours of growth arrest, cells were incubated with serum-free medium alone (control) or serum-free medium containing 10 ng/mL TGF-β1 for 72 hours. **A:** Cells were harvested, and RNA was isolated and purified. Quantitative RT-PCR was used to determine mRNA expression of *HYAL2*. **B:** Total protein was isolated and immunoblotted for total HYAL2 protein production. Densitometric analysis is shown alongside. Blots are representative of three separate experiments. **C:** Fibroblasts were seeded into eight-well glass chamber slides and grown to 60% to 70% confluence prior to growth arrest. Cells were treated with serum-free medium alone or serum-free medium containing 10 ng/mL TGF-β1. Cells were washed with phosphate-buffered saline before fixation, permeabilization, and staining to visualize HYAL2 and F-actin expression/localization. Images were captured using confocal laser scanning microscopy and are representative of three individual experiments. Total HYAL2 expression is expressed as a percentage of the total HYAL2 expression by cellular localization. **Arrowheads** indicate areas of colocalization (yellow). HYAL2 localization quantification: F-actin–stained regions (green channels) were isolated by threshold settings using ImageJ software version 1.37c to identify cytoskeleton regions of interest (ROIs). A second ROI tracing the cell boundary was selected by identifying cell membrane localization. Intensity of HYAL2 staining (red channel) in each region was expressed as a percentage fraction of the total HYAL2 staining. The remaining HYAL2 staining was identified as cytoplasmic localization. Five cells per microscopic field were assessed, and a total of three microscopic fields were analyzed per treatment condition. **D:** Fibroblasts were treated as in **B** and total HYAL2 protein was co-immunoprecipitated and subjected to immunoblot for β-actin, γ-actin, and α-smooth muscle actin (SMA). Blots are representative of three separate experiments. **E:** Cells were treated as in **C** but with additional treatment of cytochalasin B, followed by immunostaining for HYAL2 and F-actin. Images are representative of three individual experiments. Data are expressed as means ± SEM of three independent experiments. ∗∗*P* < 0.01. Scale bars = 10 μm (**C** and **E**). Original magnification: ×600 (**C**); ×400 (**E**). con., control; GADPH, glyceraldehyde phosphate dehydrogenase.

Exposure of human fibroblasts to 10 ng/mL TGF-β1 for 72 hours was previously demonstrated to induce terminal myofibroblast differentiation.^14^^,^^43^ Fibroblasts were stimulated with this established dose and duration of TGF-β1 to induce stable myofibroblast differentiation and expression of *HYAL2* mRNA assessed by qPCR. Myofibroblasts had significantly increased expression of *HYAL2* mRNA compared to undifferentiated fibroblasts (Figure 2A), and this finding was reciprocated on detection of total HYAL2 protein present in CLs (Figure 2B). The cellular localization of HYAL2 was next determined. Immunofluorescence staining for HYAL2 and filamentous actin demonstrated that in undifferentiated fibroblasts, HYAL2 was predominantly localized at the cell surface, while in differentiated myofibroblasts, HYAL2 was localized along and around the actin cytoskeleton (Figure 2C). To confirm the nature of HYAL2 association with the actin cytoskeleton in myofibroblasts in culture, the cells were assessed for co-immunoprecipitation of cytoskeletal components including β-actin, γ-actin, and α-SMA in undifferentiated and TGF-β1–differentiated myofibroblasts. The immunoblots indicated that after TGF-β1–driven myofibroblast differentiation there was increased association between HYAL2 and α-SMA (Figure 2D). To further confirm this finding, cells were treated with cytochalasin B to prevent actin filament polymerization prior to immunofluorescence for HYAL2. Disruption of the actin cytoskeleton by preventing filamentous actin polymerization also led to the disruption of cytoplasmic HYAL2, confirming that HYAL2 associates with the α-SMA cytoskeleton in myofibroblasts (Figure 2E).

### HA Catabolic Activity of Cell-Associated HYAL2 in Myofibroblasts

**Figure 3:**
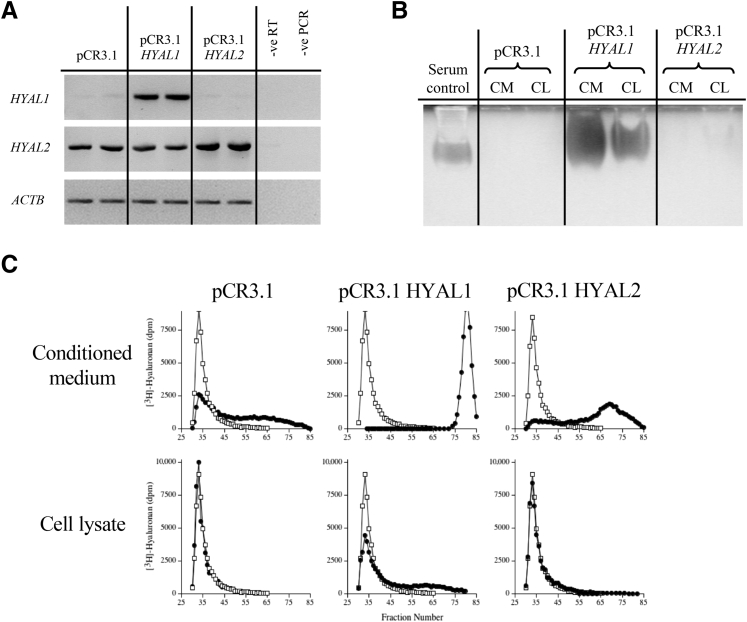
Hyaluronidase (HYAL)-2 from cell lysates (CL) of myofibroblasts has no enzymatic action in hyaluronan (HA)-degradation assays. Fibroblasts were grown to 70% confluence and were transiently transfected with pCR3.1 containing HYAL1 or HYAL2 coding regions. Control transient transfections were performed with the corresponding empty vector. Cells were growth-arrested before incubation in serum-free medium containing 10 ng/mL transforming growth factor beta 1 (TGFB1; TGF-β1). **A:** Total cellular RNA was extracted, purified, and analyzed by RT-PCR, and the products were separated on 3% agarose gels. Controls were negative (−ve) RT and negative (−ve) PCR, where no polymerase enzyme was added to the reaction mixture. **B:** HA substrate gel zymography of HYAL activity. The medium was collected, and the cells were extracted in lysis buffer. The HYAL activity was isolated from the conditioned medium (CM) and CL by DEAE-Sephacel ion exchange and lyophilized. Positive controls consisted of diluted human serum. Polyacrylamide gel electrophoresis was performed without SDS in a 7.5% polyacrylamide gel (+HA) and visualized by Alcian Blue and Coomassie Brilliant Blue stain. **C:** HYAL activity (circles) of CM and CL was further analyzed by incubating with high–molecular-weight [^3^H]-HA. HA size distribution was examined and compared with the original [^3^H]-HA preparation, incubated in the absence of the conditioned medium or CL concentrate (squares). All images are representative of four individual experiments. GADPH, glyceraldehyde phosphate dehydrogenase.

To determine the catabolic effects of HYAL2 in myofibroblasts, these cells were transiently transfected with plasmid vector (pCR3.1) containing the *HYAL2* coding region. To compare catabolic activity of HYAL2 with the catabolic activity of the other widely expressed HYAL enzyme in vertebrates, HYAL1, fibroblasts were also transfected with plasmid vector (pCR3.1) containing the *HYAL1* coding region. Semi-quantitative RT-PCR was used to examine the mRNA expression of *HYAL1* and *HYAL2* in fibroblasts transiently transfected with pCR3.1-*HYAL1* and pCR3.1-*HYAL2*. Control transfection was performed with the empty vector pCR3.1. Endogenous *HYAL1* and *HYAL2* mRNA expression was detected in control transfections. Transfection with pCR3.1-*HYAL1* resulted in increased *HYAL1* mRNA expression compared to transfection with the empty vector. Transfection with pCR3.1-*HYAL2* resulted in increased *HYAL2* mRNA expression compared to the empty vector (Figure 3A). HA substrate gel zymography of HYAL activity of fibroblasts transiently transfected with pCR3.1-*HYAL1* and pCR3.1-*HYAL2* was undertaken. Control transfections were undertaken with the empty vector pCR3.1. The HYAL activity was isolated from the CM and CL by DEAE-Sephacel ion-exchange and lyophilized. The HYAL activity was analyzed by HA-substrate gel zymography at pH 3.7. HYAL activity was represented as a clear band in the polyacrylamide gel, due to the exclusion of the Alcian Blue and Coomassie Brilliant Blue stains. Endogenous HYAL activity was not detected in the CM or CL of control transfections. Transfection with pCR3.1-*HYAL1* resulted in significant HYAL activity detected in both the CM and CL. However, transfection with pCR3.1-*HYAL2* did not result in any detectable HYAL activity in either CM or CL (Figure 3B). To confirm these results, cells transiently transfected with either pCR3.1 alone, pCR3.1-*HYAL1*, or pCR3.1-*HYAL2* were isolated from the CM and CL by DEAE-Sephacel ion-exchange chromatography and lyophilized. The CM and CL were incubated with high–molecular-weight [^3^H]-HA at pH 3.7 for 72 hours. The samples were then subjected to size-exclusion chromatography and HYAL activity assessed by comparing the elution profiles of the [^3^H]-HA incubated in the presence or absence of CM or CL. Endogenous HYAL activity was detected in the CM of the control transfections with pCR3.1 alone, partially degrading the HA to lower–molecular-weight forms. Transfection with pCR3.1-*HYAL1* increased the HYAL activity detected in the CM compared to the control transfection. The CL contained relatively less HYAL activity, only partially degrading the HA. Transfection with pCR3.1-*HYAL2* increased the HYAL activity in the CM compared to control transfections with pCR3.1 alone. HYAL activity was not detected in the CL of cells transfected with pCR3.1-*HYAL2* at all, indicating that cell-associated HYAL2 is inactive in myofibroblasts (Figure 3C).

### HYAL2 Involvement in Mediating TGF-β1–Driven Myofibroblast Differentiation

**Figure 4:**
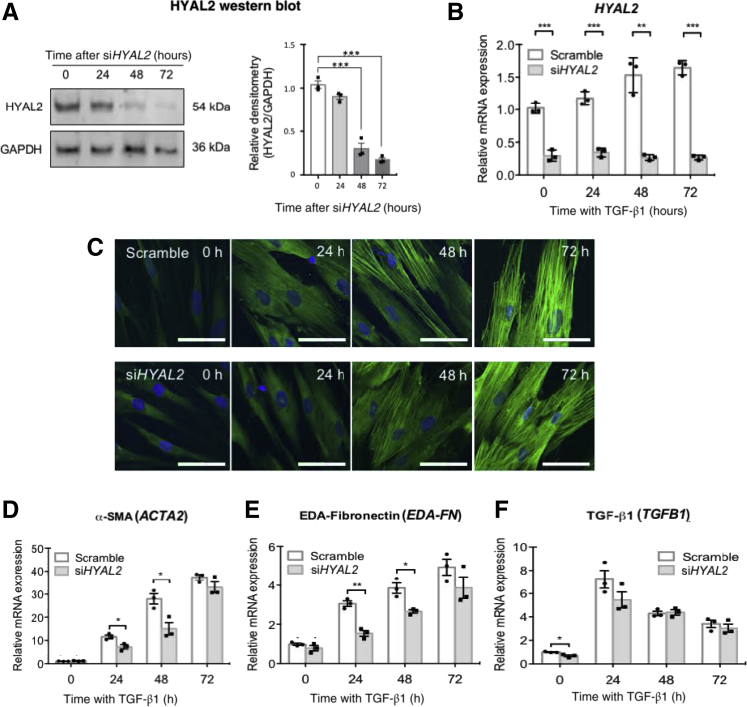
Inhibition of hyaluronidase (HYAL)-2 expression delays but does not prevent myofibroblast phenotype acquisition or transforming growth factor beta 1 (TGFB1; TGF-β1)–associated gene expression. Fibroblasts were grown to 50% confluence prior to transfection with negative control (scrambled) siRNA or with siRNA targeting *HYAL2* expression (si*HYAL2*). Cells were growth-arrested for 48 hours before treatment with serum-free medium alone or serum-free medium containing 10 ng/mL TGF-β1 for 0, 24, 48, or 72 hours. **A:** Western blot was used to assess HYAL2 protein knockdown. **B:** Quantitative RT-PCR (RT-qPCR) was used to assess *HYAL2* mRNA knockdown. **C:** Cells were then washed with phosphate-buffered saline, fixed, and permeabilized before staining to visualize α-smooth muscle actin (SMA). Images are representative of three individual experiments. **D**–**F:** RT-qPCR was also used to assess TGF-β1–regulated expression of *ACTA2* (α-SMA) (**D**), extra domain-A–fibronectin (*EDA-FN*) (**E**), and *TGFB1* (**F**) mRNA after incubation with either *HYAL2* or scrambled siRNA. Data are expressed as means ± SEM of three independent experiments. ∗*P* < 0.05, ∗∗*P* < 0.01, and ∗∗∗*P* < 0.001. Scale bars = 10 μm.

Myofibroblasts demonstrate cytoskeletal reorganization compared to undifferentiated fibroblasts. Fibroblasts generally demonstrate a narrow, spindle-shaped appearance with actin fibers forming a complex cortical meshwork at the periphery of cells. In myofibroblasts, the actin fibers coalesce to form thick parallel stress fibers that run from end to end of the cells, and the contractile protein α-SMA is incorporated into these stress fibers.^34^^,^^35^ In view of the results from Figures 1 and 2 indicating that HYAL2 demonstrated association with and relocalization to associate with the actin cytoskeleton as fibroblasts underwent differentiation to myofibroblasts, a link between HYAL2 and myofibroblast differentiation was investigated. siRNAs targeting *HYAL2* (si*HYAL2*) were used to determine the role of this protein in TGF-β1–driven myofibroblast differentiation. Successful knockdown of *HYAL2* mRNA and HYAL2 protein expression, after siRNA treatment compared with scrambled controls, was initially confirmed (Figure 4, A and B). The influence of *HYAL2* knockdown on α-SMA protein expression was subsequently assessed. There were no significant effects of si*HYAL2* transfection versus control (scrambled siRNA transfection) on α-SMA incorporation into myofibroblast stress fibers as visualized through immunofluorescence (Figure 4C). The effects of *HYAL2* knockdown on *ACTA2* (α-SMA) mRNA expression assessed by qPCR similarly demonstrated no effects on terminal myofibroblast differentiation at 72 hours (Figure 4D). Although there was a delay in increased *ACTA2* mRNA expression at 24 and 48 hours, at 72 hours after TGF-β1 stimulation *ACTA2* mRNA expression in fully differentiated myofibroblasts was comparable in si*HYAL2*-treated versus scrambled-siRNA–treated cells. EDA splice variant FN is another characteristic myofibroblast marker necessary for acquisition of this profibrotic phenotype.^48^^,^^49^ The influence of si*HYAL2* transfection on *EDA-FN* mRNA expression also demonstrated that while *HYAL2* knockdown delayed increased *EDA-FN* mRNA expression, it did not influence *EDA-FN* mRNA expression in differentiated myofibroblasts (Figure 4E). *HYAL2* knockdown also did not influence autoinduction of *TGFB1* mRNA expression in myofibroblasts, indicating that overall HYAL2 did not influence TGF-β1–dependent terminal myofibroblast differentiation (Figure 4F).

### HYAL2 Binding to RhoA and Myosin Light-Chain Kinase and Modulation of Phosphorylation of These Proteins

**Figure 5:**
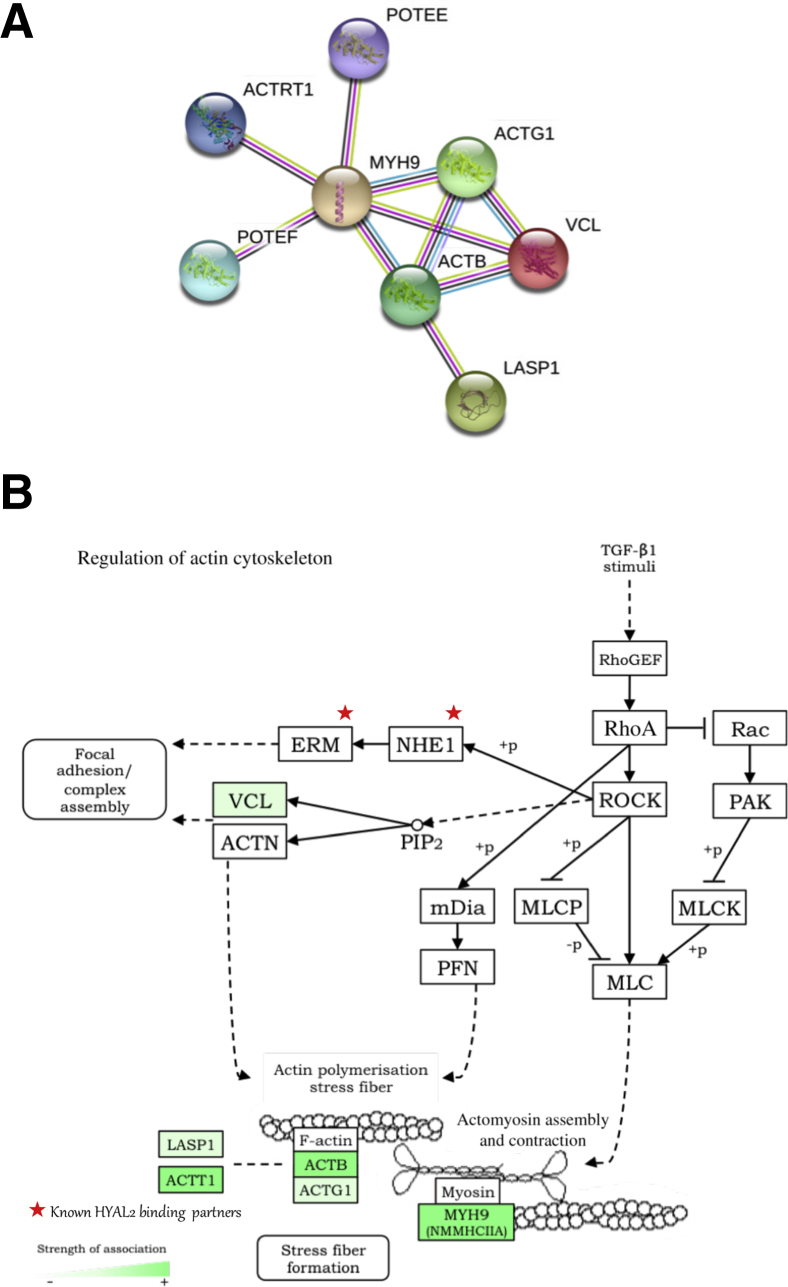
Proteins co-associated with hyaluronidase (HYAL)-2 have roles in actin organization and contraction function. **A:** Functional protein–protein association networks of proteins identified from HYAL2 co-immunoprecipitation and mass spectrometry. Protein web was created using the STRING online database version 10.5. **B:** Kyoto Encyclopedia of Genes and Genomes (KEGG) functional pathway of actin cytoskeleton organization. Depth of green indicates strength of HYAL2–protein association, as determined by tandem mass spectrometry peptide identification (where darker green indicates stronger evidence of association). Previously demonstrated HYAL2 associations are highlighted by **stars**. Pathway map was generated using KEGG Mapper version 2.8 prior to adaption. ACTB, β-actin; ACTG, γ-actin; ACTN, α-actinin; ACTRT1, actin-related protein T1; ACTT, ACT-toxin biosynthesis protein; ETV5, ETS variant transcription factor 5 [also known as Ets-related protein (ERM)]; SLC2A4 regulator, guanine–nucleotide exchange factor (also known as GEF); LASP, LIM and SH3 domain protein; mDia, mammalian diaphanous-related formin; MLC1, modulator of VRAC current 1 [also known as membrane protein (MLC)]; MYLK, myosin light chain kinase (also known as MLCK); MLCP, myosin light-chain phosphatase; MYH9, myosin heavy chain 9 (also known as MNNCHIIA); NHE, Na^+^/H^+^ exchanger; PAK, p21-activated kinase; PFN, profilin; PIP, phosphatidylinositol phosphate; POTEE, prostate, ovary, testis-expressed (POTE) ankyrin domain family member E; POTEF, POTE ankyrin domain family member F; ROCK, Rho-associated protein kinase; TGF, transforming growth factor; VCL, vinculin. Panel **B** adapted with permission from Kanehisa Laboratories.

MS was used to identify important HYAL2 cellular interactions in myofibroblasts. The Mascot score was used to justify the accuracy of protein identification, and Table 2 lists the proteins identified in descending order of significance. HYAL2 was identified as associating with actin-related cytoskeletal remodeling proteins (actin-related protein T1, β-actin, γ_1_-actin), contractile proteins (myosin heavy chain 9), prostate, ovary, testis-expressed ankyrin domain family members E/F; vinculin; and LIM and SH3 domain protein 1. Table 3 shows the GO and InterPro results from uploading the MS hits and demonstrates the lists of relevant pathways with these hits. To summarize the proteomics results into a general functional model, the known and predicted protein–protein interactions were built using the STRING database network and demonstrated that all of the proteins are closely related, providing additional evidence that HYAL2 is involved in pathways consisting of these proteins (Figure 5A). Of the suggested pathways, actin cytoskeleton regulation was selected because of the observations demonstrating that HYAL2 aligns with the cell cytoskeleton (Figure 5B). Figure 5B also demonstrates the strength of HYAL2–protein associations within that pathway. This analysis indicates a putative link between HYAL2, RhoA, and MLCK.

**Table 2:** Mass Spectrometry Mascot Scores in Descending Order of Significance

Best protein accession	Best protein description	Mascot score	Total peptides, *n* (MS and MS/MS)	Total peptides with MS/MS data, *n*	Peptide 1	Peptide 2	Peptide 3
MYH9	Myosin-9 (NMMHCIIA)	158 (e = 1.1e−011)	26	4	VVFQEFR (e = 0.018)	GDLPFVVPRR (e = 0.081)	VSHLLGINVTDFTR (e = 0.23)
MYH9	Myosin-9 (NMMHCIIA)	170 (e = 6.6e−013)	24	3	VSHLLGINVTDFTR (e = 0.0024)	VVFQEFR (e = 0.016)	RGDLPFVVPR (e = 0.024)
POTEE	POTE ankyrin domain family member E	121 (e = 5.3e−008)	13	1	SYELPDGQVITIGNER (e = 1.50 × 10^−7^)		
POTEF	POTE ankyrin domain family member F	118 (e = 1.1e−007)	12	1	SYELPDGQVITIGNER (e = 1.50 × 10^−7^)		
ACTRT1	Actin-related protein T1	101 (e = 5.3e−006)	10	1	AGLSGEIGPR (e = 0.012)		
ACTRT1	Actin-related protein T1	91 (e = 5.7e−005)	9	1	AGLSGEIGPR (e = 0.015)		
ACTB	Actin, cytoplasmic 1 (β)	132 (e = 4.2e−009)	8	1	SYELPDGQVITIGNER (e = 1.50 × 10^−7^)		
ACTG1	Actin, cytoplasmic 2 (γ)	132 (e = 4.2e−009)	8	1	SYELPDGQVITIGNER (e = 1.50 × 10^−7^)		
ACTB	Actin, cytoplasmic 1 (β)	161 (e = 5.3e−012)	7	1	SYELPDGQVITIGNER (e = 1.70 × 10^−10^)		
LASP1	Nebulin-related-LIM/SH3 domain protein 1	83 (e = 0.00031)	29				
VCL	Vinculin	61 (e = 0.057)	17

**Table 3:** GO and InterPro Results from Mass Spectrometry Hits Demonstrating Identified Relevant Pathways

Pathway ID	GO process description
GO.0070527	Platelet aggregation
GO.0001895	Retina homeostasis
GO.0030168	Platelet activation
GO.0048871	Multicellular organismal homeostasis
GO.0050878	Regulation of body fluid levels
GO.0007409	Axonogenesis
GO.0007596	Blood coagulation
GO.0048667	Cell morphogenesis and neuron differentiation
GO.0061564	Axon development


Pathway ID	GO pathway/function description
GO.0005938	Cell cortex
GO.0030863	Cortical cytoskeleton
GO.0072562	Blood microparticle
GO.0005925	Focal adhesion
GO.0031988	Membrane-bounded vesicle
GO.0015629	Actin cytoskeleton
GO.0070062	Extracellular exosome
GO.0005576	Extracellular region
GO.0071944	Cell periphery
GO.0005856	Cytoskeleton


Pathway ID	InterPro function
IPR004001	Actin, conserved site
IPR020902	Actin/actin-like conserved site
IPR004000	Actin family


Pathway ID	KEGG pathway
4810	Regulation of actin cytoskeleton
4520	Adherens junction
4670	Leukocyte transendothelial migration
4530	Tight junction
4510	Focal adhesion


KEGG, Kyoto Encyclopedia of Genes and Genomes.

**Figure 6:**
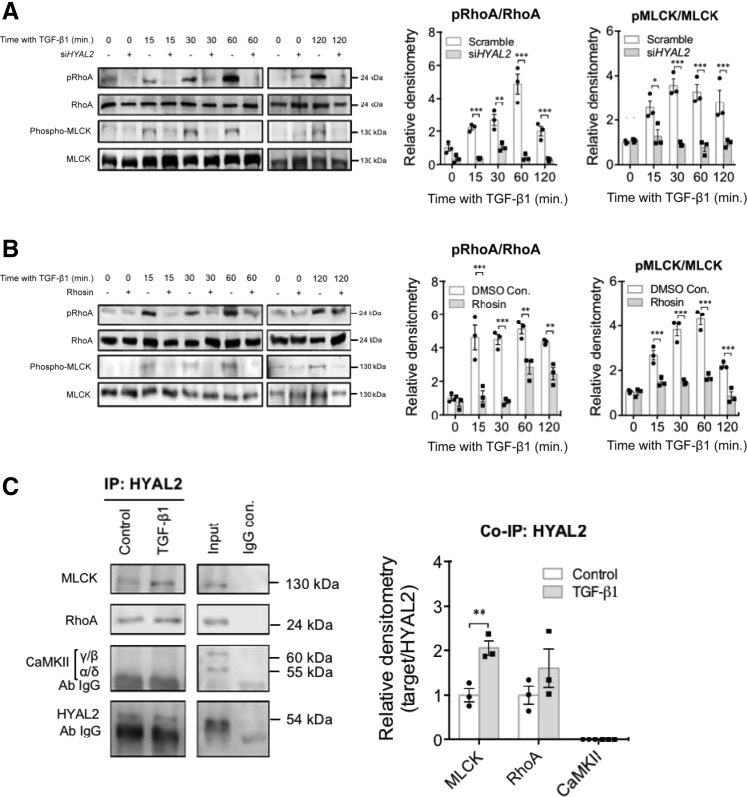
Hyaluronidase (HYAL)-2 associates with and orchestrates ras homolog family member A (RhoA) activation and downstream myosin light-chain kinase (MYLK; MLCK) activation. **A:** Fibroblasts were grown to 50% confluence prior to transfection with negative control (scramble) siRNA (−) or with siRNA targeting *HYAL2* expression (si*HYAL2*) (+). After successful transfection and growth arrest, cells were incubated with 10 ng/mL transforming growth factor (TGF)-β1 for the annotated times. At each time point, cells were harvested and protein from total cell lysate was isolated. Phosphorylation of RhoA and MLCK were determined by Western blot. Total RhoA and MLCK were used as gel loading controls. **B:** The RhoA inhibitor Rhosin was used as an inhibitor of RhoA activation. Fibroblasts were pretreated with 10 μmol/L Rhosin for 2 hours, prior to and during incubation with 10 ng/mL TGF-β1. Phosphorylation of RhoA and MLCK were determined by Western blot. **C:** Fibroblasts were grown to confluence in culture and after 48 hours of growth arrest were incubated with either serum-free medium containing 10 ng/mL TGF-β1 or serum-free medium alone (control). Anti-HYAL2 co-immunoprecipitation followed by Western blot for MLCK, RhoA, and calcium/calmodulin-dependent protein kinase type II (CaMKII) was then performed. Western blots for HYAL2 were used as loading controls. Positive (+ve) controls were total cell lysate and negative (−ve) controls were co-immunoprecipitation (IP) performed using rabbit Immunoglobulin G (IgG) in place of anti-HYAL2 antibody. All blots are representative of three independent experiments. Densitometries are expressed as the means ± SEM of three independent experiments. ∗*P* < 0.05, ∗∗*P* < 0.01, and ∗∗∗*P* < 0.001., con. control; min., minutes; p, phosphorylated.

To conclusively determine the association between the HYAL2 and RhoA pathways, fibroblasts stimulated with TGF-β1 were transfected with either si*HYAL2* or scrambled siRNA. Subsequent Western blot analysis to assess RhoA and MLCK phosphorylation was undertaken and compared to total RhoA and MLCK, respectively, as a control. The results demonstrated that *HYAL2* knockdown significantly attenuated RhoA phosphorylation at all time points after TGF-β1 stimulation. *HYAL2* knockdown also attenuated MLCK phosphorylation at 15, 30, 60, and 120 minutes after TGF-β1 stimulation. These results were confirmed by densitometric analysis of Western blot bands indicating statistically significant attenuation of RhoA and MLCK activation after *HYAL2* knockdown (Figure 6A). To determine the signaling of the link between RhoA and MLCK, a chemical inhibitor of RhoA (Rhosin) was used in subsequent experiments. The data indicated that Rhosin effectively attenuated RhoA phosphorylation. Furthermore, RhoA inhibition with Rhosin also abrogated MLCK phosphorylation at 15, 30, 60, and 120 minutes after TGF-β1 stimulation, and this was confirmed on densitometric analysis (Figure 6B). Co-immunoprecipitation was used to examine any direct interaction between these proteins, and the results indicated that HYAL2 interacts directly with RhoA in both fibroblasts and myofibroblasts. HYAL2 also interacts with MLCK, and this interaction is increased after stimulation with TGF-β1. Previous work has demonstrated an important role for calcium/calmodulin-dependent protein kinase type II signaling in mediating TGF-β1–driven fibroblast to myofibroblast differentiation.^42^ This study therefore also tested for direct interactions between HYAL2 and calcium/calmodulin-dependent protein kinase type II and identified no association between these two proteins (Figure 6C).

### Attenuated Expression of RhoA-Dependent Genes after *HYAL2* Knockdown

**Figure 7:**
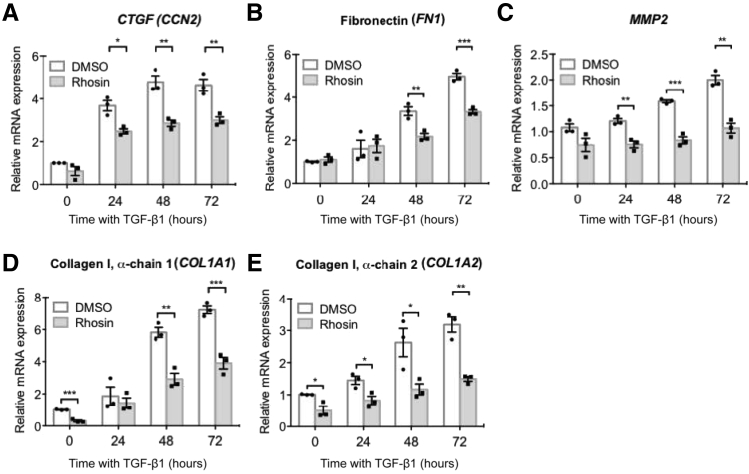
Ras homolog family member A (RhoA) regulates cellular communication network factor 2 [CCN2, also known as connective tissue growth factor (CTGF)], fibronectin, matrix metallopeptidase 2 [MMP2, also known as matrix metalloproteinase (MMP)-2], and collagen I gene expression. Fibroblasts were grown to confluent monolayers on 35-mm tissue culture plates. They were then growth-arrested and pretreated with either dimethyl sulfoxide (DMSO) alone (as a control) or with 10 μmol/L Rhosin for 2 hours, prior to incubation with 10 ng/mL transforming growth factor beta 1 (TGFB1, also known as TGF-β1) for 0, 24, 48, or 72 hours. Quantitative RT-PCR was used to assess the effects of RhoA inhibition on mRNA expression of *CTGF* (**A**), fibronectin 1 (*FN1*) (**B**), *MMP2* (**C**), collagen type I alpha 1 chain (*COL1A1*) (**D**), and collagen type I alpha 2 chain (*COL1A2*) (**E**). Data are expressed as means ± SEM of three independent experiments. ∗*P* < 0.05, ∗∗*P* < 0.01, and ∗∗∗*P* < 0.001.

**Figure 8:**
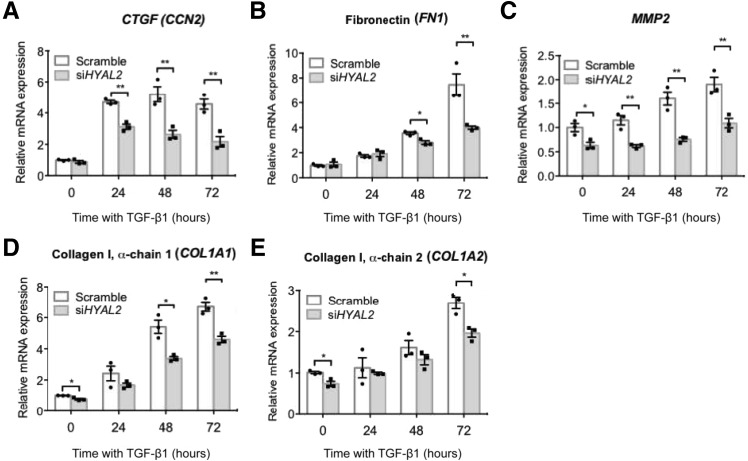
Ras homolog family member A (RhoA)-dependent gene expression is attenuated by hyaluronidase [HYAL2, also known as (HYAL)-2] knockdown. Fibroblasts were grown to 50% confluence prior to transfection with negative control (scramble) siRNA or with siRNA targeting HYAL2 expression (si*HYAL2*). Cells were subsequently growth-arrested before treatment with serum-free medium containing 10 ng/mL transforming growth factor beta 1 [TGFB1, also known as (TGF)-β1], for 0, 24, 48, or 72 hours. Quantitative RT-PCR was used to assess the effects of *HYAL2* knockdown on mRNA expression of cellular communication network factor 2 (*CTGF*) (**A**), fibronectin 1 (*FN1*) (**B**), matrix metallopeptidase 2 (**C**), collagen type I alpha 1 chain (*COL1A1*) (**D**), and collagen type I alpha 2 chain (*COL1A2*) (**E**). Data are expressed as means ± SEM of three independent experiments. ∗*P* < 0.05, ∗∗*P* < 0.01.

**Figure 9:**
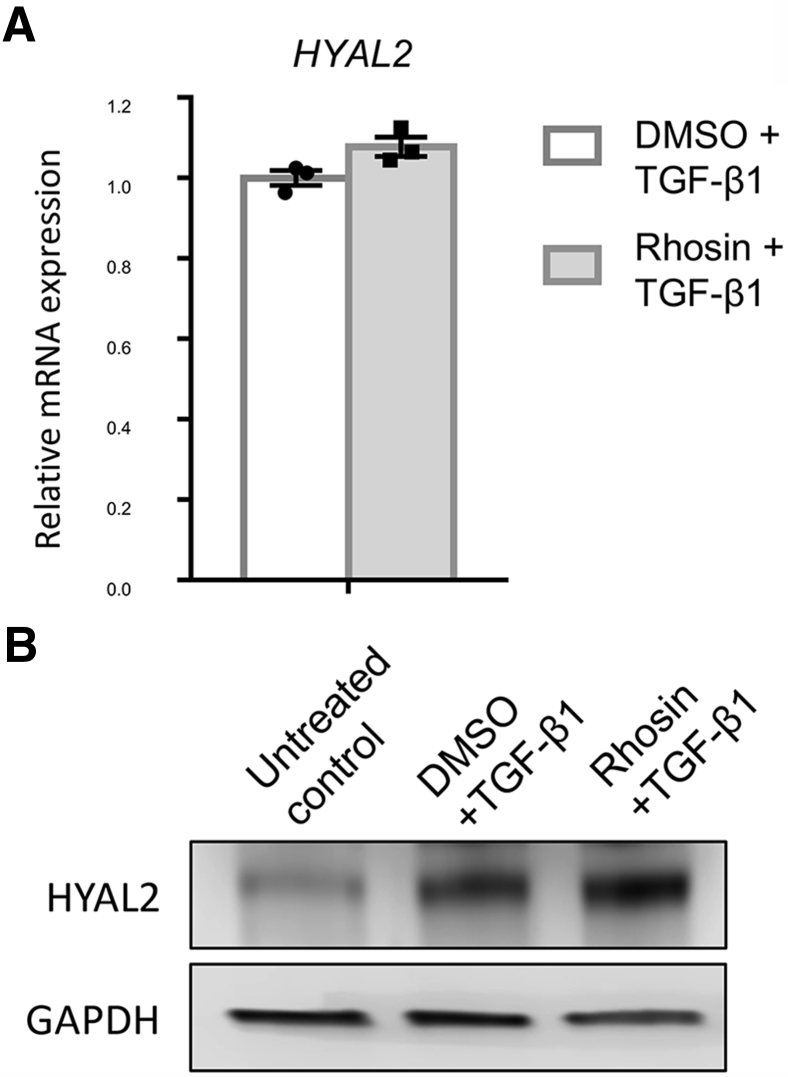
Ras homolog family member A (RhoA) inhibition does not attenuate hyaluronidase 2 (*HYAL2*) mRNA or hyaluronidase (HYAL)-2 protein expression. Fibroblasts were grown to confluent monolayers on 35-mm tissue culture plates. They were then growth-arrested and pretreated with either dimethyl sulfoxide (DMSO) alone (as a control) or with 10 μmol/L Rhosin for 2 hours, prior to and during incubation with 10 ng/mL transforming growth factor beta 1 [TGFB1, also known as (TGF)-β1] for 72 hours. **A:** Quantitative RT-PCR was used to assess the effect of RhoA inhibition on *HYAL2* mRNA expression. **B:** Western blot was used to assess the effect of RhoA inhibition on HYAL2 protein expression. GAPDH, glyceraldehyde phosphate dehydrogenase.

RhoA is a small GTPase protein in the Rho family, which has been implicated in regulating several fibrogenic genes including collagens, FNs, matrix metalloproteinases (MMPs), and connective tissue growth factor [CTGF; also known as cellular communication network factor (CCN2)].^50^, ^51^, ^52^, ^53^, ^54^ To confirm the involvement of RhoA in the regulation of these genes in our experimental cell systems, fibroblasts were incubated with 10 ng/mL TGF-β1 for up to 72 hours to induce myofibroblast differentiation and treated with either a chemical RhoA inhibitor (Rhosin) or incubated with dimethyl sulfoxide alone. The effects of RhoA inhibition on mRNA expression of *CCN2*, *FN1*, *MMP2*, and *COL1A1*/*COL1A2* (collagen type 1 α chains) were subsequently assessed using qPCR. The results demonstrated that RhoA inhibition attenuated *CTGF*, *FN1*, *MMP2*, *COL1A1*, and *COL1A2* mRNA expression, indicating that RhoA regulates expression of these genes in myofibroblasts (Figure 7). The effects of *HYAL2* knockdown on these RhoA-regulated genes was then investigated (Figure 8). *CTGF* mRNA expression was significantly attenuated upon *HYAL2* knockdown at all timepoints after TGF-β1 stimulation (Figure 8A). Expression levels of *FN1*, *MMP2*, *COL1A1*, and *COL1A2* all demonstrated significant attenuation after *HYAL2* knockdown compared to scrambled controls in cells that had differentiated to myofibroblasts (Figure 8, B–E). Furthermore, there was no effect on *HYAL2* mRNA (Figure 9A) or HYAL2 protein (Figure 9B) expression after Rhosin treatment, suggesting that RhoA inhibition did not directly influence HYAL2 expression.

### HYAL2 Regulation of RhoA-Mediated Contractility and Migration in Myofibroblasts

**Figure 10:**
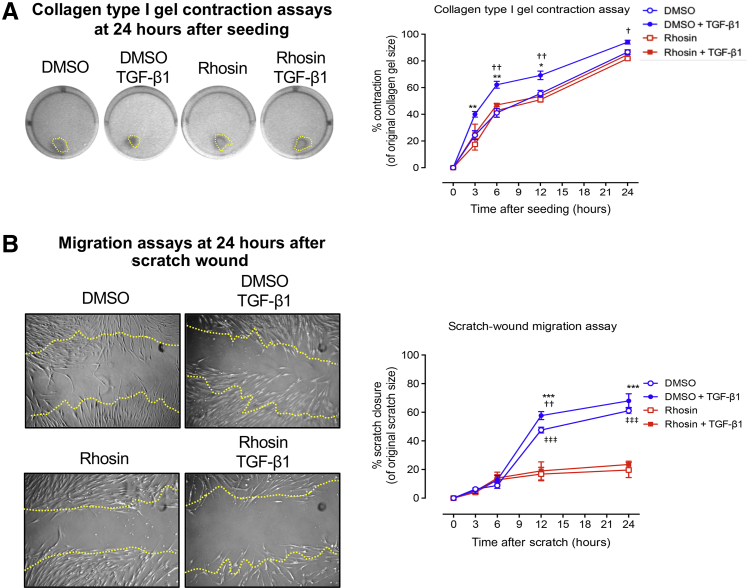
Ras homolog family member A (RhoA) regulates transforming growth factor beta 1 (TGFB1, also known as TGF-β1)–dependent contractility and migration in myofibroblasts. Fibroblasts were grown to confluent monolayers, growth-arrested for 48 hours, and then treated with 10 μmol/L Rhosin for 2 hours prior to and during TGF-β1 incubation. **A:** Cells were seeded into collagen gels and left to polymerize before incubation in serum-free medium alone or serum-free medium containing 10 ng/mL TGF-β1. Collagen gels were imaged over the annotated time points and measured for analysis of rate of contraction. **Dotted lines** indicate the measured gel areas at this time point. **B:** Cells were growth-arrested and scratched using a 20 μL pipette tip. After a phosphate-buffered saline wash, the medium was replaced with fresh serum-free medium or serum-free medium containing 10 ng/mL TGF-β1. Scratch assays were imaged at the indicated time points, and the area of closure was measured to assess rate of migration. **Dotted lines** represent the original scratch edges. Data are expressed as means ± SEM of three individual experiments. ∗*P* < 0.05, ∗∗*P* < 0.01, and ∗∗∗*P* < 0.001 control + TGF-β1 versus treated + TGF-β1; ^†^*P* < 0.05, ^††^*P* < 0.01 control versus treated samples; ^‡‡‡^*P* < 0.001 control versus control + TGF-β1 samples. DMSO, dimethyl sulfoxide.

**Figure 11:**
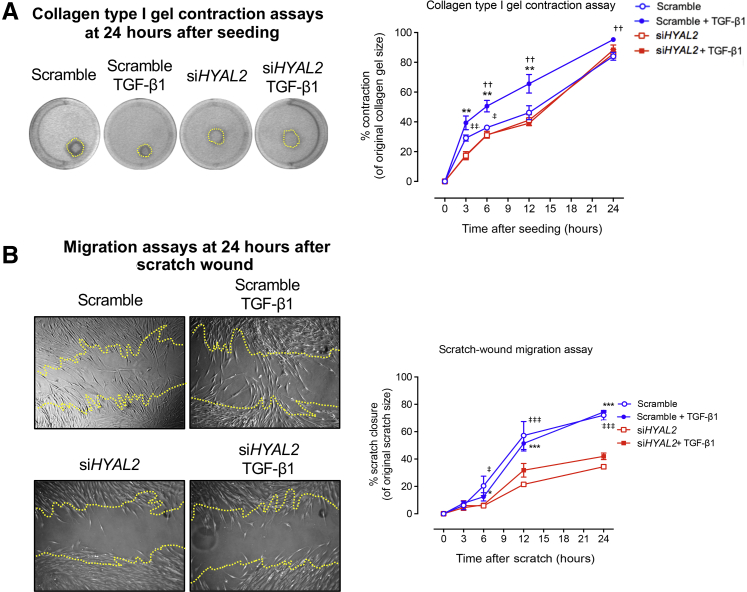
Hyaluronidase (HYAL2, also known as HYAL-2) mediates Ras homolog family member A (RhoA)-dependent contractility and migration in myofibroblasts. Fibroblasts were grown to 50% confluence prior to transfection with negative control (scramble) siRNA or with siRNA targeting *HYAL2* expression (si*HYAL2*). **A:** Cells were seeded into collagen gels and left to polymerize before incubation in serum-free medium alone or serum-free medium containing 10 ng/mL transforming growth factor (TGF)-β1. Collagen gels were imaged over the annotated time points and measured for analysis of rate of contraction. **Lines** indicate the measured gel areas at this time point. **B:** Cells were growth-arrested and scratched using a 20-μL pipette tip. After a phosphate-buffered saline wash, the medium was replaced with fresh serum-free medium or serum-free medium containing 10 ng/mL TGF-β1. Scratch assays were imaged at the indicated time points, and the area of closure was measured to assess the rate of migration. **Lines** represent the original scratch edges. Data are expressed as the means ± SEM of three individual experiments. ∗*P* < 0.05, ∗∗*P* < 0.01, and ∗∗∗*P* < 0.001 control + TGF-β1 versus treated + TGF-β1; ^††^*P* < 0.01 control versus treated samples; ^‡^*P* < 0.05, ^‡‡^*P* < 0.01, ^‡‡‡^*P* < 0.001 control versus control + TGF-β1 samples.

The activity of Rho GTPases in regulating the polymerization and organization of actin and myosin filaments has been previously established.^52^ The role of RhoA in regulating cytoskeletal-related myofibroblast functions was therefore assessed in our experimental cell systems. The effect of RhoA on TGF-β1–driven activated fibroblast contractility was initially assessed. The results demonstrated that TGF-β1 enhanced collagen type I gel contraction, whereas treatment with the RhoA inhibitor (Rhosin) abrogated TGF-β1–driven activated fibroblast contractility (Figure 10A). In addition, RhoA inhibition also markedly attenuated TGF-β1–driven migration in these cells (Figure 10B). To determine the role of HYAL2 in regulating TGF-β1–driven activated fibroblast contractility and migration, fibroblasts were transfected with either scrambled siRNA or si*HYAL2* and subsequently incubated with 10 ng/mL TGF-β1. Similar to the effects seen with RhoA inhibition, knockdown of *HYAL2* reduced TGF-β1-driven fibroblast contractility and significantly suppressed migration, over the course of 24 hours (Figure 11). Thus, RhoA and HYAL2 are both involved in mediating pathways associated with TGF-β1–driven activated fibroblast contractility and migration.

## Discussion

HYAL2 is a 54-kDa protein that has high-level expression in most tissues, including skin, liver, kidneys, skeleton, heart, and lungs, suggesting that it has an important biological role. However, while the primary function of HYAL2 is considered to be enzymatic, it is only a weak HA-degrading enzyme. Hence, although recent studies have indicated that mutations in HYAL2 lead to significant cardiac, skeletal, hematopoietic, and other abnormalities,^15^^,^^16^^,^^18^^,^^20^ the cellular mechanisms through which HYAL2 achieves its effects and regulates biology are poorly understood.

The findings from this study, in conjunction with other recently published studies, indicate that HYAL2 has important and previously undescribed nonenzymatic effects that influence distinct and opposing fibroblastic cell functions. Previous studies demonstrated the role of nuclear HYAL2 as a key regulator of *CD44* alternative splicing, and in particular, highlighted its function in promoting the expression of an antifibrotic CD44 splice variant termed CD44v7/8.^27^^,^^43^ Cell-surface expression of this CD44 splice variant in fibroblasts resulted in prevention and reversal of TGF-β1–driven myofibroblast differentiation, thereby limiting profibrotic tissue damage. In contrast, the results presented here demonstrate that cytoplasmic HYAL2 promotes profibrotic processes. This study shows that TGF-β1 promotes HYAL2 relocalization to the cytoplasm, where it binds to the actin cytoskeleton. HYAL2 in this context modulates RhoA signaling, promoting downstream profibrotic myofibroblast functions. Specifically, HYAL2 enhances RhoA-dependent cell migration and, to a lesser extent, contractility. Many studies demonstrate RhoA to be promigratory, while some studies report RhoA to be antimigratory.^52^^,^^55^^,^^56^ While this dichotomy is not fully understood, the findings from this study indicate that HYAL2–RhoA interactions are crucial in regulating RhoA-dependent migration and may explain its divergent functions. HYAL2 also promotes increased collagen, FN, CTGF (CCN2) and MMP2 expression. In these studies, despite having low HA catabolic activity, HYAL2 influences wide-ranging cellular and matrix effects through its influence on myofibroblast phenotype and function. Given the central role of myofibroblasts in regulating homeostasis and driving pathology, the broad expression of HYAL2 in tissues and the widespread impact of its dysregulation on homeostasis and disease are now more understandable. Whether HYAL2 has similar nonenzymatic actions in nonstromal cell populations remains to be investigated.

HYAL2 requires a pH optimum of 4.0 for its HA-breakdown activity.^22^ Little is known regarding the factors that may govern the switch from enzymatic to nonenzymatic HYAL2 functions. At the cell surface, glycosylphosphatidylinositol-linked HYAL2 has been shown to interact with the Na^+^/H^+^ exchanger 1.^57^ Na^+^/H^+^ exchanger 1 has been proposed to create acidic microenvironments at the cell surface accounting for any membrane-bound HYAL2 catalytic activity. Furthermore, cell-surface HYAL2 catalytic activity has previously been found to be dependent on the expression of cell-surface CD44.^58^ Nuclear HYAL2 has been found to be associated with a range of RNA- and DNA-processing proteins and influences the incorporation of serine- and arginine-rich splice factors and snRNAs into the spliceosome.^27^ HYAL2 has also been identified intracellularly within lysosomes and endosomes and now binding to the cytoskeleton.^43^^,^^59^ At each of these cell locations, HYAL2 was identified as having distinct cellular functions, and it is possible to speculate that the distinct actions of HYAL2 are influenced by its cellular localization. It may be that the nonenzymatic functions of HYAL2 predominate when the protein is localized in a nonacidic microenvironment and detached from CD44 in support of findings from previous studies.^58^ The mechanisms that influence HYAL2 cellular localization are as yet unknown, and the protein–protein interactions that may influence HYAL2 trafficking between the cell surface, cytoplasm, and nucleus will be the focus of further study.

HYAL2 is identified as having a catalytic domain, an HA-binding domain, a glycosylphosphatidylinositol-anchor domain, and an epidermal growth factor–like domain. However, the other putative functional domains of HYAL2 that may regulate the recently identified nonenzymatic functions remain undescribed. It is known that HYAL2 is involved in a number of protein–protein interactions, including HYAL2–CD44 coupling, HYAL2–WW domain–containing oxidoreductase 1 and HYAL2–cell migration–inducing and HA-binding protein interactions.^23^^,^^24^^,^^60^ In these studies, HYAL2 interactions with these particular proteins influence the effects of HYAL2, subsequently influencing distinct cellular functions. As an example, HYAL2 interaction with cell migration–inducing and HA-binding protein (formerly known as KIAA1199) is thought to enhance the catalytic activity of HYAL2.^60^ Hence, it is also possible that as-yet–unidentified proteins may influence a conformational change in HYAL2 structure that is crucial for its various nonenzymatic actions. In the field of fibrosis research, the generation of catalytically inactive mutants of HYAL2, and an understanding of both HYAL2 structural biology and the distinct peptide domains and HYAL2–protein interactions that could promote antifibrotic versus profibrotic cellular functions, offer an important area of research.

In this study we demonstrated that while cytoskeletal-linked HYAL2 did not mediate TGF-β1–driven myofibroblast differentiation, it regulated some very important myofibroblast functions that ultimately influence fibrogenesis. Migration and contractility are crucial myofibroblast functions facilitating scarring.^61^ Increased generation of collagens and FNs are hallmarks of fibrotic matrix production by myofibroblasts. In addition, numerous studies have established a link between increased expression of CTGF (CCN2) and MMP2 in fibrotic disease.^53^^,^^62^ All of these functions are downstream effects of RhoA signaling, as indicated in the experiments using the RhoA inhibitor Rhosin.^51^^,^^54^ Given the observed direct interaction between HYAL2 and RhoA demonstrated in this study, it appears that cytoskeletal-linked HYAL2 is involved in the orchestration of RhoA signaling and is likely to influence all downstream RhoA effects. Furthermore, as there seems to be a basal interaction between HYAL2 and RhoA, even in resting/unstimulated fibroblasts, it is speculated that after TGF-β1 stimulation, both HYAL2 and RhoA move together from the cell membrane to the cytoskeleton, where they can both associate with and activate MLCK. In conclusion, HYAL2 in myofibroblasts associates with the actin cytoskeleton and interacts with cytoskeletal-associated RhoA, and this interaction is essential for mediating downstream profibrotic RhoA-signaling effects.

## References

[1] Kosaki R., Watanabe K., Yamaguchi Y. (1999). Overproduction of hyaluronan by expression of the hyaluronan synthase Has2 enhances anchorage-independent growth and tumorigenicity. Cancer Res.

[2] Legg J.W., Lewis C.A., Parsons M., Ng T., Isacke C.M. (2002). A novel PKC-regulated mechanism controls CD44 ezrin association and directional cell motility. Nat Cell Biol.

[3] Itano N., Atsumi F., Sawai T., Yamada Y., Miyaishi O., Senga T., Hamaguchi M., Kimata K. (2002). Abnormal accumulation of hyaluronan matrix diminishes contact inhibition of cell growth and promotes cell migration. Proc Natl Acad Sci U S A.

[4] Ito T., Williams J.D., Al-Assaf S., Phillips G.O., Phillips A.O. (2004). Hyaluronan and proximal tubular cell migration. Kidney Int.

[5] Camenisch T.D., Schroeder J.A., Bradley J., Klewer S.E., McDonald J.A. (2002). Heart-valve mesenchyme formation is dependent on hyaluronan-augmented activation of ErbB2-ErbB3 receptors. Nat Med.

[6] Zoltan-Jones A., Huang L., Ghatak S., Toole B.P. (2003). Elevated hyaluronan production induces mesenchymal and transformed properties in epithelial cells. J Biol Chem.

[7] Brecht M., Mayer U., Schlosser E., Prehm P. (1986). Increased hyaluronate synthesis is required for fibroblast detachment and mitosis. Biochem J.

[8] Tammi R., Tammi M. (1991). Correlations between hyaluronan and epidermal proliferation as studied by [3H]glucosamine and [3H]thymidine incorporations and staining of hyaluronan on mitotic keratinocytes. Exp Cell Res.

[9] Evanko S.P., Angello J.C., Wight T.N. (1999). Formation of hyaluronan- and versican-rich pericellular matrix is required for proliferation and migration of vascular smooth muscle cells. Arterioscler Thromb Vasc Biol.

[10] Krolikoski M., Monslow J., Pure E. (2019). The CD44-HA axis and inflammation in atherosclerosis: a temporal perspective. Matrix Biol.

[11] Johnson P., Arif A.A., Lee-Sayer S.S., Dong Y. (2018). Hyaluronan and its interactions with immune cells in the healthy and inflamed lung. Front Immunol.

[12] Theocharis A.D., Manou D., Karamanos N.K. (2019). The extracellular matrix as a multitasking player in disease. FEBS J.

[13] Meran S., Steadman R. (2011). Fibroblasts and myofibroblasts in renal fibrosis. Int J Exp Pathol.

[14] Jenkins R.H., Thomas G.J., Williams J.D., Steadman R. (2004). Myofibroblastic differentiation leads to hyaluronan accumulation through reduced hyaluronan turnover. J Biol Chem.

[15] Andre B., Duterme C., Van Moer K., Mertens-Strijthagen J., Jadot M., Flamion B. (2011). Hyal2 is a glycosylphosphatidylinositol-anchored, lipid raft-associated hyaluronidase. Biochem Biophys Res Commun.

[16] Chowdhury B., Hemming R., Hombach-Klonisch S., Flamion B., Triggs-Raine B. (2013). Murine hyaluronidase 2 deficiency results in extracellular hyaluronan accumulation and severe cardiopulmonary dysfunction. J Biol Chem.

[17] Chowdhury B., Xiang B., Liu M., Hemming R., Dolinsky V.W., Triggs-Raine B. (2017). Hyaluronidase 2 deficiency causes increased mesenchymal cells, congenital heart defects, and heart failure. Circ Cardiovasc Genet.

[18] Muggenthaler M.M., Chowdhury B., Hasan S.N., Cross H.E., Mark B., Harlalka G.V., Patton M.A., Ishida M., Behr E.R., Sharma S., Zahka K., Faqeih E., Blakley B., Jackson M., Lees M., Dolinsky V., Cross L., Stanier P., Salter C., Baple E.L., Alkuraya F.S., Crosby A.H., Triggs-Raine B., Chioza B.A. (2017). Mutations in HYAL2, encoding hyaluronidase 2, cause a syndrome of orofacial clefting and cor triatriatum sinister in humans and mice. PLoS Genet.

[19] Albeiroti S., Ayasoufi K., Hill D.R., Shen B., de la Motte C.A. (2015). Platelet hyaluronidase-2: an enzyme that translocates to the surface upon activation to function in extracellular matrix degradation. Blood.

[20] Petrey A.C., Obery D.R., Kessler S.P., Flamion B., de la Motte C.A. (2016). Hyaluronan depolymerization by megakaryocyte hyaluronidase-2 is required for thrombopoiesis. Am J Pathol.

[21] Colombaro V., Jadot I., Decleves A.E., Voisin V., Giordano L., Habsch I., Malaisse J., Flamion B., Caron N. (2015). Lack of hyaluronidases exacerbates renal post-ischemic injury, inflammation, and fibrosis. Kidney Int.

[22] Stern R. (2003). Devising a pathway for hyaluronan catabolism: are we there yet?. Glycobiology.

[23] Duterme C., Mertens-Strijthagen J., Tammi M., Flamion B. (2009). Two novel functions of hyaluronidase-2 (Hyal2) are formation of the glycocalyx and control of CD44-ERM interactions. J Biol Chem.

[24] Hsu L.J., Schultz L., Hong Q., Van Moer K., Heath J., Li M.Y., Lai F.J., Lin S.R., Lee M.H., Lo C.P., Lin Y.S., Chen S.T., Chang N.S. (2009). Transforming growth factor beta1 signaling via interaction with cell surface Hyal-2 and recruitment of WWOX/WOX1. J Biol Chem.

[25] Miller A.D. (2008). Hyaluronidase 2 and its intriguing role as a cell-entry receptor for oncogenic sheep retroviruses. Semin Cancer Biol.

[26] Rai S.K., Duh F.M., Vigdorovich V., Danilkovitch-Miagkova A., Lerman M.I., Miller A.D. (2001). Candidate tumor suppressor HYAL2 is a glycosylphosphatidylinositol (GPI)-anchored cell-surface receptor for jaagsiekte sheep retrovirus, the envelope protein of which mediates oncogenic transformation. Proc Natl Acad Sci U S A.

[27] Midgley A.C., Oltean S., Hascall V., Woods E.L., Steadman R., Phillips A.O., Meran S. (2017). Nuclear hyaluronidase 2 drives alternative splicing of CD44 pre-mRNA to determine profibrotic or antifibrotic cell phenotype. Sci Signal.

[28] Green F.H. (2002). Overview of pulmonary fibrosis. Chest.

[29] Chapman H.A. (2004). Disorders of lung matrix remodeling. J Clin Invest.

[30] Eddy A.A. (2000). Molecular basis of renal fibrosis. Pediatr Nephrol.

[31] Bedossa P., Paradis V. (2003). Liver extracellular matrix in health and disease. J Pathol.

[32] Anversa P., Li P., Zhang X., Olivetti G., Capasso J.M. (1993). Ischaemic myocardial injury and ventricular remodelling. Cardiovasc Res.

[33] Francis G.S., McDonald K., Chu C., Cohn J.N. (1995). Pathophysiologic aspects of end-stage heart failure. Am J Cardiol.

[34] Desmouliere A., Geinoz A., Gabbiani F., Gabbiani G. (1993). Transforming growth factor-beta 1 induces alpha-smooth muscle actin expression in granulation tissue myofibroblasts and in quiescent and growing cultured fibroblasts. J Cell Biol.

[35] Vaughan M.B., Howard E.W., Tomasek J.J. (2000). Transforming growth factor-beta1 promotes the morphological and functional differentiation of the myofibroblast. Exp Cell Res.

[36] Evans R.A., Tian Y.C., Steadman R., Phillips A.O. (2003). TGF-beta1-mediated fibroblast-myofibroblast terminal differentiation-the role of Smad proteins. Exp Cell Res.

[37] Meran S., Thomas D., Stephens P., Martin J., Bowen T., Phillips A., Steadman R. (2007). Involvement of hyaluronan in regulation of fibroblast phenotype. J Biol Chem.

[38] Webber J., Meran S., Steadman R., Phillips A. (2009). Hyaluronan orchestrates transforming growth factor-beta1-dependent maintenance of myofibroblast phenotype. J Biol Chem.

[39] Webber J., Jenkins R.H., Meran S., Phillips A., Steadman R. (2009). Modulation of TGFbeta1-dependent myofibroblast differentiation by hyaluronan. Am J Pathol.

[40] Simpson R.M., Meran S., Thomas D., Stephens P., Bowen T., Steadman R., Phillips A. (2009). Age-related changes in pericellular hyaluronan organization leads to impaired dermal fibroblast to myofibroblast differentiation. Am J Pathol.

[41] Bommaya G., Meran S., Krupa A., Phillips A.O., Steadman R. (2011). Tumour necrosis factor-stimulated gene (TSG)-6 controls epithelial-mesenchymal transition of proximal tubular epithelial cells. Int J Biochem Cell Biol.

[42] Midgley A.C., Rogers M., Hallett M.B., Clayton A., Bowen T., Phillips A.O., Steadman R. (2013). Transforming growth factor-beta1 (TGF-beta1)-stimulated fibroblast to myofibroblast differentiation is mediated by hyaluronan (HA)-facilitated epidermal growth factor receptor (EGFR) and CD44 co-localization in lipid rafts. J Biol Chem.

[43] Midgley A.C., Duggal L., Jenkins R., Hascall V., Steadman R., Phillips A.O., Meran S. (2015). Hyaluronan regulates bone morphogenetic protein-7-dependent prevention and reversal of myofibroblast phenotype. J Biol Chem.

[44] Shevchenko A., Jensen O.N., Podtelejnikov A.V., Sagliocco F., Wilm M., Vorm O., Mortensen P., Shevchenko A., Boucherie H., Mann M. (1996). Linking genome and proteome by mass spectrometry: large-scale identification of yeast proteins from two dimensional gels. Proc Natl Acad Sci U S A.

[45] Perkins D.N., Pappin D.J., Creasy D.M., Cottrell J.S. (1999). Probability-based protein identification by searching sequence databases using mass spectrometry data. Electrophoresis.

[46] Szklarczyk D., Morris J.H., Cook H., Kuhn M., Wyder S., Simonovic M., Santos A., Doncheva N.T., Roth A., Bork P., Jensen L.J., von Mering C. (2017). The STRING database in 2017: quality-controlled protein-protein association networks, made broadly accessible. Nucleic Acids Res.

[47] Cawston T.E., Barrett A.J. (1979). A rapid and reproducible assay for collagenase using [1-14C]acetylated collagen. Anal Biochem.

[48] Tomasek J.J., Gabbiani G., Hinz B., Chaponnier C., Brown R.A. (2002). Myofibroblasts and mechano-regulation of connective tissue remodelling. Nat Rev Mol Cell Biol.

[49] Hinz B. (2006). Masters and servants of the force: the role of matrix adhesions in myofibroblast force perception and transmission. Eur J Cell Biol.

[50] Guo F., Debidda M., Yang L., Williams D.A., Zheng Y. (2006). Genetic deletion of Rac1 GTPase reveals its critical role in actin stress fiber formation and focal adhesion complex assembly. J Biol Chem.

[51] Li C., Zhen G., Chai Y., Xie L., Crane J.L., Farber E., Farber C.R., Luo X., Gao P., Cao X., Wan M. (2016). RhoA determines lineage fate of mesenchymal stem cells by modulating CTGF-VEGF complex in extracellular matrix. Nat Commun.

[52] Etienne-Manneville S., Hall A. (2002). Rho GTPases in cell biology. Nature.

[53] Sakai N., Nakamura M., Lipson K.E., Miyake T., Kamikawa Y., Sagara A., Shinozaki Y., Kitajima S., Toyama T., Hara A., Iwata Y., Shimizu M., Furuichi K., Kaneko S., Tager A.M., Wada T. (2017). Inhibition of CTGF ameliorates peritoneal fibrosis through suppression of fibroblast and myofibroblast accumulation and angiogenesis. Sci Rep.

[54] Sun K., Duan X., Cai H., Liu X., Yang Y., Li M., Zhang X., Wang J. (2016). Curcumin inhibits LPA-induced invasion by attenuating RhoA/ROCK/MMPs pathway in MCF7 breast cancer cells. Clin Exp Med.

[55] Jatho A., Hartmann S., Kittana N., Mugge F., Wuertz C.M., Tiburcy M., Zimmermann W.H., Katschinski D.M., Lutz S. (2015). RhoA ambivalently controls prominent myofibroblast characteristics by involving distinct signaling routes. PLoS One.

[56] Guo S.J., Zhang P., Wu L.Y., Zhang G.N., Chen W.D., Gao P.J. (2016). Adenovirus-mediated overexpression of septin 2 attenuates alpha-smooth muscle actin expression and adventitial myofibroblast migration induced by angiotensin II. J Vasc Res.

[57] Bourguignon L.Y., Singleton P.A., Diedrich F., Stern R., Gilad E. (2004). CD44 interaction with Na+-H+ exchanger (NHE1) creates acidic microenvironments leading to hyaluronidase-2 and cathepsin B activation and breast tumor cell invasion. J Biol Chem.

[58] Harada H., Takahashi M. (2007). CD44-dependent intracellular and extracellular catabolism of hyaluronic acid by hyaluronidase-1 and -2. J Biol Chem.

[59] Lepperdinger G., Strobl B., Kreil G. (1998). HYAL2, a human gene expressed in many cells, encodes a lysosomal hyaluronidase with a novel type of specificity. J Biol Chem.

[60] Yoshida H., Nagaoka A., Kusaka-Kikushima A., Tobiishi M., Kawabata K., Sayo T., Sakai S., Sugiyama Y., Enomoto H., Okada Y., Inoue S. (2013). KIAA1199, a deafness gene of unknown function, is a new hyaluronan binding protein involved in hyaluronan depolymerization. Proc Natl Acad Sci U S A.

[61] Darby I.A., Zakuan N., Billet F., Desmouliere A. (2016). The myofibroblast, a key cell in normal and pathological tissue repair. Cell Mol Life Sci.

[62] Mansour S.G., Puthumana J., Coca S.G., Gentry M., Parikh C.R. (2017). Biomarkers for the detection of renal fibrosis and prediction of renal outcomes: a systematic review. BMC Nephrol.

